# Challenges and new technologies in adoptive cell therapy

**DOI:** 10.1186/s13045-023-01492-8

**Published:** 2023-08-18

**Authors:** Pengchao Zhang, Guizhong Zhang, Xiaochun Wan

**Affiliations:** 1 Center for Protein and Cell-based Drugs, Institute of Biomedicine and Biotechnology, Shenzhen Institute of Advanced Technology, Chinese Academy of Sciences, 1068 Xueyuan Avenue, Nanshan District, Shenzhen, 518055 People’s Republic of China; 2 University of Chinese Academy of Sciences, Beijing, 100049 People’s Republic of China

**Keywords:** Adoptive cell therapy, Chimeric antigen receptor, T cell receptor, Cancer therapy, Gene transduction

## Abstract

Adoptive cell therapies (ACTs) have existed for decades. From the initial infusion of tumor-infiltrating lymphocytes to the subsequent specific enhanced T cell receptor (TCR)-T and chimeric antigen receptor (CAR)-T cell therapies, many novel strategies for cancer treatment have been developed. Owing to its promising outcomes, CAR-T cell therapy has revolutionized the field of ACTs, particularly for hematologic malignancies. Despite these advances, CAR-T cell therapy still has limitations in both autologous and allogeneic settings, including practicality and toxicity issues. To overcome these challenges, researchers have focused on the application of CAR engineering technology to other types of immune cell engineering. Consequently, several new cell therapies based on CAR technology have been developed, including CAR-NK, CAR-macrophage, CAR-γδT, and CAR-NKT. In this review, we describe the development, advantages, and possible challenges of the aforementioned ACTs and discuss current strategies aimed at maximizing the therapeutic potential of ACTs. We also provide an overview of the various gene transduction strategies employed in immunotherapy given their importance in immune cell engineering. Furthermore, we discuss the possibility that strategies capable of creating a positive feedback immune circuit, as healthy immune systems do, could address the flaw of a single type of ACT, and thus serve as key players in future cancer immunotherapy.

**Supplementary Information:**

The online version contains supplementary material available at 10.1186/s13045-023-01492-8

## Introduction

Currently, immunotherapy methods based on immune checkpoint inhibitors, tumor vaccines, and adoptive cell therapy (ACT) have revolutionized cancer treatment. Immunotherapy has advantages over the three conventional therapies (surgery, radiotherapy, and chemotherapy) in that it can stimulate the immune system to permanently eradicate residual or disseminated tumor cells and restore immune function that has been weakened by radiotherapy and chemotherapy [1, 2]. Following extensive clinical research, immunotherapy has demonstrated promising application potential in the management of various malignancies, with the potential to enhance therapeutic effect, prolong patient survival, and improve patient quality of life [2, 3].

ACT has recently received considerable attention due to the remarkable success of chimeric antigen receptor (CAR)-T cell therapy in the treatment of hematological malignancies. In contrast to chemotherapy, ACT is an active biological strategy that employs “live” drugs, whereby patient immune cells are collected, expanded, and engineered in vitro before being reinfused into the patient’s body to kill pathogens and/or cancer cells. Tumor-infiltrating lymphocyte (TIL) therapy, engineered T cell receptor (TCR)-T cell therapy, and CAR-T cell therapy are the three primary ACTs. Among them, CAR-T therapy has received marketing approval and achieved considerable success in the treatment of hematological malignancies [4]. Additionally, researchers have expressed interest in the application of CAR engineering techniques to modify other immune cells. Consequently, a series of new ACTs based on CAR technology have been developed, including CAR-natural killer (NK), CAR-macrophage (M), CAR-γδT, and CAR-natural killer T (NKT). Clinical studies involving these methods are rapidly increasing, despite their advantages and drawbacks.

In this review, we provide an overview of several ACTs of current research interest, including TIL, TCR-T, CAR-T, CAR-NK, CAR-M, CAR-γδT, and CAR-NKT therapies. We discuss the advantages and challenges (and their potential solutions) of these therapies as well as the research being conducted in these areas. ACTs frequently involve the operation of gene transduction, which is often difficult for primary immune cells. Therefore, we also provide a summary of the numerous gene transduction techniques used in ACTs.

## ACTs

### TIL: founder of ACT

TILs are a group of lymphocytes that infiltrate into tumors, including T cells, NK cells, and others. These lymphocytes can recognize and destroy tumor cells as well as mobilize bystander immune cells to help combat the tumor. However, the lack of sufficient TILs and the dysfunction produced by the unfavorable tumor microenvironment (TME) frequently prevent TILs from performing their anti-tumor activity as well as they could. TIL therapy, a method based on TIL isolation, ex vivo expansion, and subsequent re-implantation, was developed and is being attempted to treat cancer.

#### Advantages

**Fig. 1:**
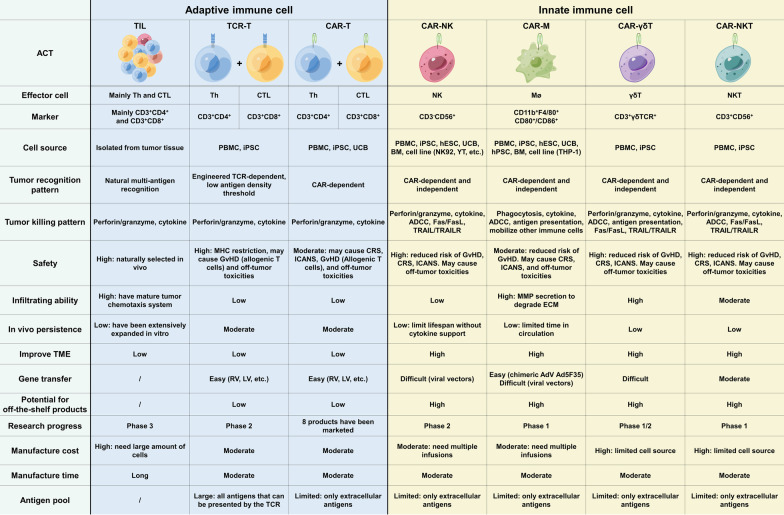
Summary of current adoptive cell therapies in cancer treatment. Th, helper T cell; CTL, cytotoxic T lymphocyte; PBMC, peripheral blood mononuclear cell; iPSC, induced pluripotent stem cell; hESC, human embryonic stem cell; UCB, umbilical cord blood; hPSC, human pluripotent stem cell; BM, bone marrow; ADCC, antibody-dependent cell-mediated cytotoxicity; CRS, cytokine release syndrome; ICANS, immune effector cell-associated neurotoxicity syndrome; GvHD, graft versus host disease; MMP, matrix metalloproteinase; ECM, extracellular matrix; RV, retrovirus; LV, lentivirus; and AdV, adenovirus

TIL therapy offers several unique advantages for treating solid tumors (Fig. 1): (1) TILs can circumvent the problem of heterogeneity of solid tumors because they are composed of T cells that target multiple antigens in cancer cells. (2) TILs, which are isolated from tumors, can easily infiltrate tumors because they already possess an appropriate chemokine receptor system. (3) As TILs are derived from patients, the reinfused TILs typically do not cause noticeable adverse effects, and no studies have reported off-target effects or cytokine release syndrome (CRS) in TIL therapy thus far.

#### Research progress

**Fig. 2:**
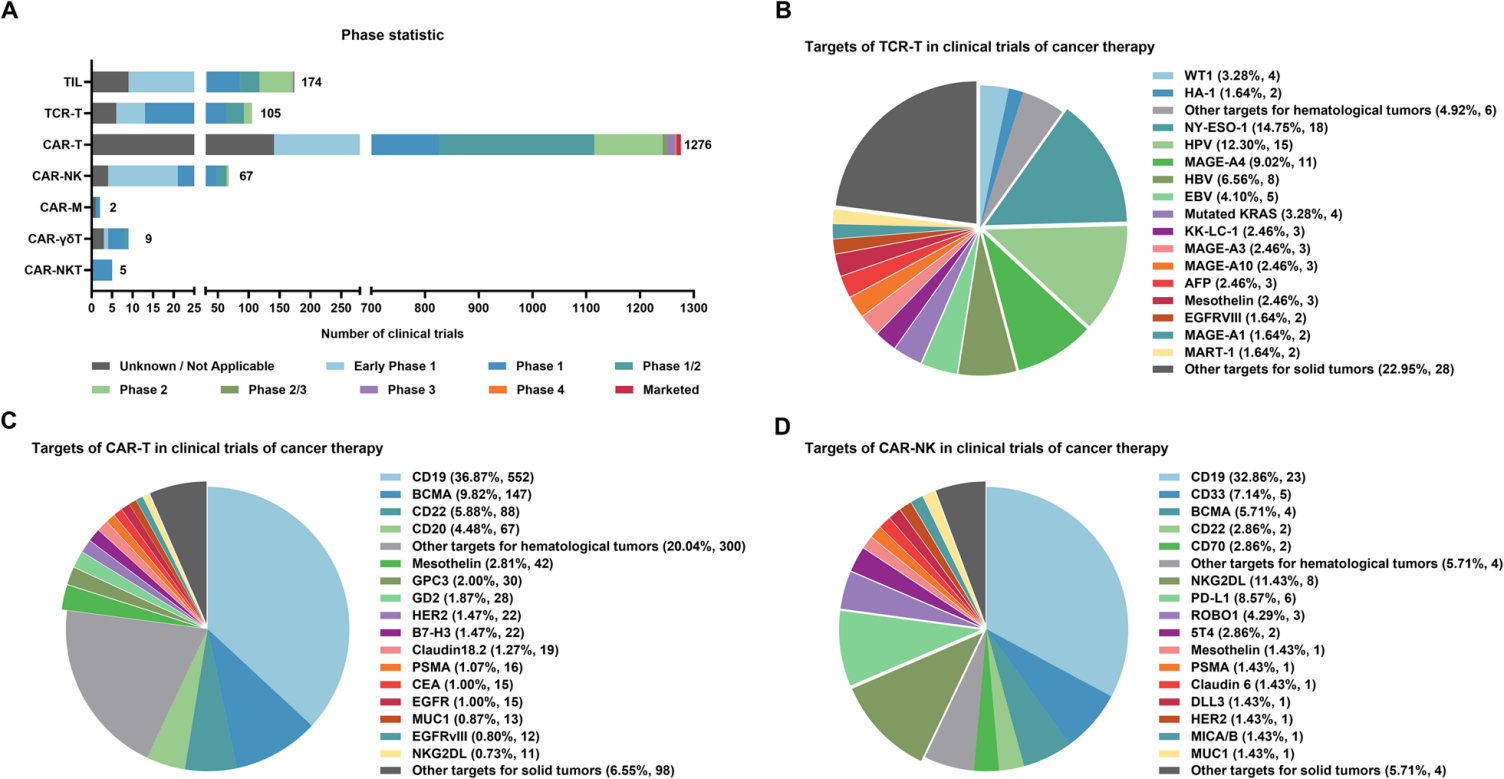
Development stages of adoptive cell therapies and targets of TCR-T, CAR-T, and CAR-NK cells in clinical trials of cancer therapy. **A** The developmental stage of each adoptive cell therapy was counted. **B**–**D** Targets of TCR-T (**B**), CAR-T (**C**), and CAR-NK (**D**) in clinical trials of cancer therapy. The top targets were highlighted separately in the pie chart; low proportion or unknown targets were merged into the other targets section. Each target of the multi-target CAR was counted separately. Data were obtained from clinicaltrials.gov and were updated as of July 2023. WT1, Wilms tumor 1; HA-1, minor histocompatibility antigen 1; HPV, human papillomavirus; MAGE, melanoma-associated antigen; HBV, hepatitis B virus; EBV, Epstein–Barr virus; AFP, alpha-fetoprotein; BCMA, B cell maturation antigen; GPC3, glypican-3; GD2, disialoganglioside; PSMA, prostate-specific membrane antigen; CEA, carcinoembryonic antigen; NKG2DL, NKG2D ligand; DLL3, delta-like ligand 3; and MICA/B, major histocompatibility complex class I-related chain A/B

The use of autologous TILs in ACT to elicit tumor regression in patients with metastatic malignant melanoma was first demonstrated by Rosenberg et al. in 2002 [5]. Since then, numerous studies on TIL treatment have been conducted (Fig. 2A and Additional file 1), yielding encouraging clinical results. In three clinical trials for melanoma, Rosenberg et al. discovered that the objective response rate (ORR) of TIL treatment ranged from 49–72%, with 28% of patients achieving complete response (CR) [6]. Similarly, a phase 2 clinical trial utilizing TIL therapy for melanoma in 2016 revealed an ORR of 56% and a CR rate of 24% [7]. Additionally, TIL treatment was also shown to drive the regression of metastatic cervical cancer in a phase 2 clinical investigation (ORR = 33%) (NCT01585428) [8].

#### Challenges and potential solutions

TIL therapy has yet to be approved by the US Food and Drug Administration (FDA) owing to its numerous limitations (Figs. 1 and 2A). First, not all tumor tissues are suitable for isolating active lymphocytes that target tumor cells. Second, because a large number of lymphocytes are needed for TIL therapy, some patients with rapidly progressing diseases cannot wait for the isolated TIL to grow in vitro, which typically takes 2 months. Furthermore, TILs become exhausted from prolonged in vitro expansion, showing poor cytotoxicity and persistence [9]. Finally, because TIL therapy is entirely customized and cannot yield a universal product, it is challenging to maintain consistent TIL quality. To address the problem of T cell exhaustion, a new generation of TIL therapies has emerged, aiming to genetically modify TILs to improve their persistence and anticancer activity. For instance, it has been demonstrated in preclinical research that knocking out *programmed cell death 1* (*PDCD1)* using gene editing technologies, such as clustered regularly interspaced short palindromic repeats-associated protein 9 (CRISPR/Cas9) or transcription activator-like effector nucleases (TALENs), can improve the anti-tumor effect of TIL therapy [10]. However, no solutions currently exist for the other limitations of TIL therapy. Nonetheless, TIL therapy is essential for demonstrating the anticancer potential of immune cell adoptive transfer therapy.

The limitations of TIL therapy have prompted researchers to explore more practical therapies. Scientists have discovered that TILs from different patients can recognize the same antigens that are highly expressed in tumor cells, such as MART-1 and glycoprotein 100 (gp100) [11]. A series of cell therapies were developed as a result of these discoveries, which brought classical TIL therapy into the era of precision targeting.

### TCR-T: a sharp sword against solid tumors

TCR-T cell therapy is a process wherein normal T cells are transduced with antigen-specific TCR α and β chains to produce tumor-specific T cells, which are then amplified and reinfused into the body to specifically kill tumor cells. According to the clinical trial findings of Rosenberg et al. [12], genetically modified lymphocytes expressing multiple TCRs against specific tumor antigens (TCR-engineered T cells) have promising therapeutic potential for metastatic melanoma. Two of the 15 patients with melanoma in the trial demonstrated objective regression. This is the first study to show that TCR-T cells are feasible for cancer therapy, even though the result is not satisfactory.

#### Advantages

Although TCR-T cells are artificially created, their TCR structure is derived from naturally occurring T cells; consequently, they preserve many of the advantages of natural TCRs (Fig. 1). (1) With a complete TCR structure, TCR-T cells can fully mediate TCR signaling and recruit all costimulatory molecules and thus show potent anti-tumor activity. (2) TCR activation is dependent on the antigen presented by major histocompatibility complex (MHC). Because MHC can present endogenous overexpressed antigens and neoantigens as well as foreign viral proteins and is unaffected by the subcellular localization of such antigens, TCR-T cell therapy is effective against a large target antigen pool. (3) Only a very small amount of target antigen is required to activate TCR, allowing engineered TCR-T cells to maintain their cytotoxicity against target cells with low antigen density [13].

#### Research progress

TCR-T cell therapy has recently received increased attention, and many studies are progressing to clinical trials as a result. Approximately 100 clinical trials of TCR-T cell therapy have been conducted thus far, although the majority are still in phase 1 or phase 2 (Fig. 2A and Additional file 1). Based on the advantages of TCR-T cell therapy, it has been clinically tested in many solid tumors such as melanoma, hepatocellular carcinoma, lung cancer, and cervical cancer. Robbins et al. reinfused NY-ESO-1 targeted TCR-T cells into 18 and 20 patients with synovial sarcoma and malignant melanoma, in 2015, and reported ORRs of 61% and 55%, respectively (NCT00670748) [14]. Furthermore, Rapoport et al. administered TCR-T cells targeting NY-ESO-1 to 20 patients with multiple myeloma, and 16 (80%) of them achieved clinical response (NCT01352286) [15].

#### Challenges and potential solutions

Although TCR-T cell therapy has achieved promising results in clinical trials, preclinical research and industrialization still face many challenges (Fig. 1). Consequently, clinical research on TCR-T cell therapy is advancing slower than expected.

1. On-target off-tumor toxicity

The ideal cancer therapeutic target should have three important characteristics: tumor specificity, high immunogenicity, and high expression in tumor tissues [16]. Currently, TCR-T cell therapy focuses on three types of targets: (1) tumor-associated antigens (TAAs) that are highly expressed by tumor cells but not or only weakly expressed by normal cells, such as cancer-testis antigen [14], melanoma-associated antigen-4 (MAGE-A4), and mesothelin (Fig. 2B). However, TAA expression in some critical tissues cannot be entirely ruled out in practice, which frequently leads to intolerable toxicity. Otherwise, T cells undergo thymus negative selection, which eliminates lymphocytes with high affinity for self-antigens, resulting in the affinity of TCR targeting TAAs being low. Both of these limitations restrict the anti-tumor effectiveness of TCR-T cell therapy targeting TAAs. (2) Oncoviral antigens, such as E6 and E7 proteins of HPV16 (Fig. 2B), which are linked to vulvar, vaginal, and cervical cancer, can be naturally presented on the cell surface [17, 18], making them effective tumor therapeutic antigens. However, the applicability of such antigens is limited because human cancers caused by oncoviruses account for only 12% [19]. (3) Neoantigens generated by mutations [20], such as KRAS (G12V) and KRAS (G12D) mutant (Fig. 2B), which are closely related to pancreatic and colorectal cancers [21], are promising therapeutic targets. However, these neoantigens are rarely found in the natural world, and the technology for screening neoantigenic epitopes has not yet been developed [17].

Targeting inappropriate antigens with T cells can lead to serious and sometimes fatal toxicities, which have been observed in clinical trials. In 2009, Johnson et al. reinfused 20 and 16 patients with melanoma with TCR-T cells targeting MART-1 and gp100, respectively [22]. Although 30% and 19% of the patients achieved objective regression, the normal melanocytes of the skin, eyes, and ears were also attacked, causing serious harm to patients. A more serious case reported in 2013 was the trial conducted by Morgan et al. wherein MAGE-A3 (KVAELVHFL) was the target [23]. In this study, TCR-T cells also recognized the MAGE-A12 (KMAELVHFL) in the nervous system, which directly contributed to the deaths of two patients. These cases suggest that selecting an appropriate target is essential for ensuring the safety and efficacy of TCR-T cell therapy. Further research regarding the selection of tumor target antigens is required.

2. Poor persistence

Before reinfusion, TCR-T cells should be expanded in vitro to a sufficient quantity, which often induces terminal differentiation [24]. Consequently, TCR-T cells frequently fail to mount a durable immune response in patients [9]. The major approaches currently used to address this issue involve either eliminating Treg and other immunosuppressive cells from the body or producing TCR-T cells using less differentiate T cells such as naïve T cells (Tn), stem cell-like memory T cells (Tscm), and central memory T cells (Tcm) [25].

3. Expression and correct pairing of engineered TCR

The expression and assembly of engineered TCR may be affected by the competition between exogenous and endogenous TCR [26]. Studies have shown that the expression and activity of engineered TCR are improved after the endogenous TCR is precisely knocked down by small interfering RNAs (siRNA) [27]. Furthermore, because TCRs are heterodimers of α and β chains, there is a risk that the endogenous TCR chain may be mispaired with the exogenous chain. This might have undesirable effects, such as a reduction in the quantity and function of correctly paired exogenous TCR or, more seriously, the attacking of normal cells by mispaired TCR that has not undergone thymic negative selection [28]. Mispaired TCR-induced toxicity has been observed in vitro and in mouse models [29, 30]. However, this issue can be resolved by murinizing the constant region of exogenous TCR to avoid pairing with endogenous TCR [26], or by introducing more cysteine residues to the constant region of exogenous TCR α and β chains to increase their pairing efficiency [31]. Cloning engineered TCR into γδT cells may also be an effective approach because they do not express TCR α and β chains [32, 33].

4. Unable to handle the constraints of the TME

The TME has a substantial impact on TCR-T cell function. In solid tumors, the expression of CXCL9 and other chemokines that recruit T cells is decreased, whereas the concentration of inhibitory cells and molecules is increased, creating an inhibitory immune milieu that substantially inhibits TCR-T cell activity [34]. Combining immune checkpoint inhibitors may help address this issue. In a mouse lung cancer model, Moon et al. combined TCR-T cells with PD-1 antibody and observed a significant increase in the anti-tumor efficacy of the TCR-T cells [35]. Additionally, the hypoxic and acidic environment present in solid tumors also constrains the function of TCR-T cells. An antacid drug called omeprazole can relieve the low pH environment present in tumors [36], which may increase the in vivo anti-tumor activity of TCR-T cells.

Clinical studies using TCR-T cell therapy have mainly focused on treating solid tumors, including melanoma, colon cancer, and synovial cell sarcoma, and have shown promising outcomes. However, some of the problems with this therapy require attention. Finding specific targets remains a crucial task in this area. Simultaneously, similar to other immune cell therapies, TCR-T cell therapy must also overcome challenges such as the immunosuppressive TME and poor infiltration and persistence. These challenges severely restrict the use of TCR-T cell therapy in clinical settings.

### CAR-T: great success, but many challenges remain

T cells can recognize antigens as peptides presented by MHC via TCR; however, tumor cells frequently downregulate the expression of MHC-I molecules to evade T cell recognition. CAR was developed to circumvent this constraint. Currently, the application potential of T cell therapy has increased due to the development of CAR-T cell therapy. Similar to TCR-T cell therapy, CAR-T cell therapy uses gene transduction techniques (retrovirus, lentivirus, non-viral vector, etc.) to confer T cells the ability to precisely attack tumors by introducing antigen-specific CAR molecules into them. However, because of differences in their structural makeup and antigen-recognition mechanisms, CAR and TCR function differently.

#### Structure of CAR

**Fig. 3:**
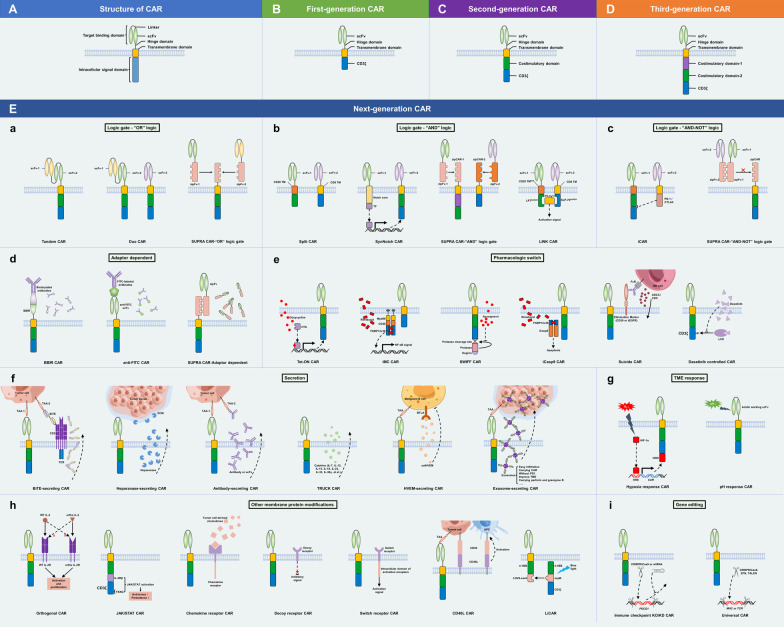
Basic structure of CAR and the evolution of CAR design. **A** The basic CAR structure consists of the extracellular antigen recognition domain (typically scFv), hinge domain, transmembrane domain, and one or more intracellular signaling domains. **B** The first-generation CARs contain a scFv for single-antigen recognition and subsequent ligation of the hinge domain, a transmembrane domain, and an intracellular CD3ζ for activation signal transmission. **C**, **D** The second- and third-generation CARs introduce one and two costimulatory domains separately, usually 4-1BB or CD28, to enhance the proliferation and persistence of CAR-T cells. **E** Many next-generation CARs have been developed to further enhance the anti-tumor potential of CAR-T cells, mainly including “OR” logic-gated CARs to improve antigen recognition profile **a** “AND” **b** “AND-NOT” **c** logic-gated CARs to improve recognition specificity, adaptor-dependent (**d**) and pharmacologic switch CARs (**e**) to enhance controllability, secretion CARs with enhanced anti-tumor ability (**f**), TME response CARs (**g**), other modifications of membrane proteins (**h**), and gene editing CARs (**i**). scFv, single-chain fragment variable; TM, transmembrane domain; TF, transcription factor; BBIR, biotin-binding immune receptor; rtTA, reverse tetracycline transcriptional activator; ADCC, antibody-dependent cellular cytotoxicity; CDC, complement-depended cytotoxicity; solHVEM, soluble herpes virus entry mediator; HRE, hypoxia response elements; and ODD, oxygen-dependent degradation domain

The function of CAR-T cells is determined by the structure of the CAR molecule, which, in contrast to that of TCR, is designed rather than produced naturally. The following are the four main components of CAR (Fig. 3A):

1. Extracellular target-binding domain

This domain, which can employ a single-chain variable fragment (scFv), nanobody, or ligand of the target, confers targeting specificity to CAR-T cells. In addition to specificity, the binding affinity of this domain to the target is another key factor affecting how well a CAR performs. Overly low or high affinities will not produce the desired results [37]. Moreover, charge density [38], epitope location [39], and target antigen density [40] must also be considered while designing a CAR.

2. Hinge domain

The hinge domain is located extracellularly and serves as a link connecting the target-binding domain with the transmembrane domain. Recent studies have indicated that it also has an impact on CAR function. Because the target may be located proximally or distally to the plasma membrane, the hinge length can affect the binding of the CAR molecule to the target at different spatial positions [41]. Therefore, CAR molecules should be created with an appropriate hinge length for the specific target antigens.

3. Transmembrane domain

The main function of the transmembrane domain is to anchor the CAR to the plasma membrane. However, recent studies have shown that it can also have an impact on the expression and stability of CAR, affect the formation of immune synapse, and be associated with the dimerization of endogenous signaling molecules [42, 43].

4. Intracellular signal domain

This domain employs both signal transduction and costimulatory domains to transmit activation signals to T cells. The signal transduction domain is typically CD3ζ or immunoglobulin Fc receptor FcεRIγ, which contains immunoreceptor tyrosine activation motifs and can thus mimic the signal transduction function of TCR. Costimulatory domains are usually derived from the CD28 receptor family (CD28, ICOS) or the tumor necrosis factor receptor family (4-1BB, OX40, CD27) and can synergize costimulatory molecules to enhance intracellular activation signals [44, 45].

#### Development of CAR

A number of CAR-based cell therapies have been developed to date with the ongoing optimization of CAR structure. Here, we use CAR-T cell therapy as the representative to introduce how CAR-based cell therapy has evolved (Fig. 3).

1. First-generation CAR

Eshhar was a pioneer in developing the first generation of CAR-T cell therapy. In 1997, he implanted T cells with a specific scFv coupled to intracellular CD3ζ, the prototype of CAR (Fig. 3B), to bypass the MHC restrictions of TCR. However, the therapeutic application of the first-generation CAR-T was not ideal because T cells often become exhausted due to a lack of costimulatory signals [46].

2. Second-generation CAR

Second-generation CAR was developed by June et al., who introduced 4-1BB as a costimulatory domain into first-generation CAR (Fig. 3C). The eight CAR-T cell therapies that have been made commercially available thus far are all second-generation therapies (Table 1). Recent findings have demonstrated that patients treated with second-generation CD19 CAR-T cells not only achieve complete remission but also maintain CAR-T cells in vivo for up to 10 years following therapy, demonstrating that the costimulatory domain has greatly improved the persistence of CAR-T cells [47]. The ongoing presence of CAR-T cells in vivo facilitates long-term monitoring and elimination of tumor cells.

3. Third-generation CAR

**Table 1:** Marketed CAR-T cell therapies

Name	Target	Manufacturer	Country	Disease	ORR	CRR	Adverse event of any grade (grade ≥ 3)	CRS of any grade (grade ≥ 3)	NT of any grade (grade ≥ 3)	Approved time	PMID
Kymriah (Tisagenlecleucel)	CD19	Novartis pharma	America	B-ALL	81%	60%	100% (88%)	77% (46%)	40% (13%)	2017 Aug	29385370
				LBCL (3rd-line treatment)	52%	40%	100% (89%)	58% (22%)	26% (15%)	2018 May	30501490
				FL (3rd-line treatment)	86%	69%	99% (78%)	49% (0%)	37% (3%)	2022 May	34921238
Yescarta (Axicabtagene ciloleucel)	CD19	Kite pharma	America	LBCL (3rd-line treatment)	82%	58%	100% (95%)	93% (13%)	64% (28%)	2017 Oct	29226797
				FL (3rd-line treatment)	92%	74%	99% (86%)	82% (7%)	59% (19%)	2021 Mar	34895487
				LBCL (2nd-line treatment)	83%	65%	100% (91%)	92% (6%)	60% (21%)	2022 Apr	34891224
Tecartus (Brexucabtagene autoleucel)	CD19	Kite pharma	America	MCL	93%	67%	100% (98%)	91% (15%)	63% (31%)	2020 Jul	32242358
				B-ALL	71%	56%	100% (95%)	89% (24%)	60% (25%)	2021 Oct	34097852
Breyanzi (Lisocabtagene maraleucel)	CD19	Juno therapeutics/bristol myers squibb	America	LBCL (3rd-line treatment)	73%	53%	99% (79%)	42% (2%)	30% (10%)	2021 Feb	32888407
				LBCL (2nd-line treatment)	86%	66%	N.A. (92%)	49% (1%)	12% (4%)	2022 Jun	35717989
Abecma (Idecabtagene vicleucel)	BCMA	Bristol myers squibb/bluebird bio	America	MM (5th-line treatment)	73%	33%	100% (99%)	84% (5%)	18% (3%)	2021 Mar	33626253
Carteyva (Relmacabtagene autoleucel)	CD19	JW therapeutics	China	LBCL (3rd-line treatment)	78%	53%	92% (56%)	48% (5%)	20% (3%)	2021 Sep	36842849
Carvykti (Ciltacabtagene autoleucel)	BCMA	Janssen biotech/legend biotech	America	MM (5th-line treatment)	97%	67%	100% (94%)	95% (4%)	21% (9%)	2022 Feb	34175021
CT103A (Equecabtagene autoleucel)	BCMA	IASO biotherapeutics/innovent biologics	China	MM (4th-line treatment)	96%	74%	N.A. (N.A.)	93% (1%)	2% (0%)	2023 Jun	33512480, 35314675

Third-generation CAR contains two costimulatory domains, such as CD28/4-1BB and CD28/OX40 (Fig. 3D). However, although third-generation CAR-T cell therapy outperformed second-generation CAR-T cell therapy in terms of persistence and amplification, it failed to demonstrate superior anti-tumor activity in vivo against hematologic malignancies [48–50]. Therefore, second-generation therapies remain the most commonly utilized therapies. These studies also demonstrate that, at least in the treatment of hematologic tumors, the addition of costimulatory molecules does not always result in increased CAR function. However, third-generation CAR may prove advantageous in the treatment of solid tumors, which contain complicated microenvironments that severely limit T cell activation and persistence.

4. Next-generation CAR

**Fig. 4:**
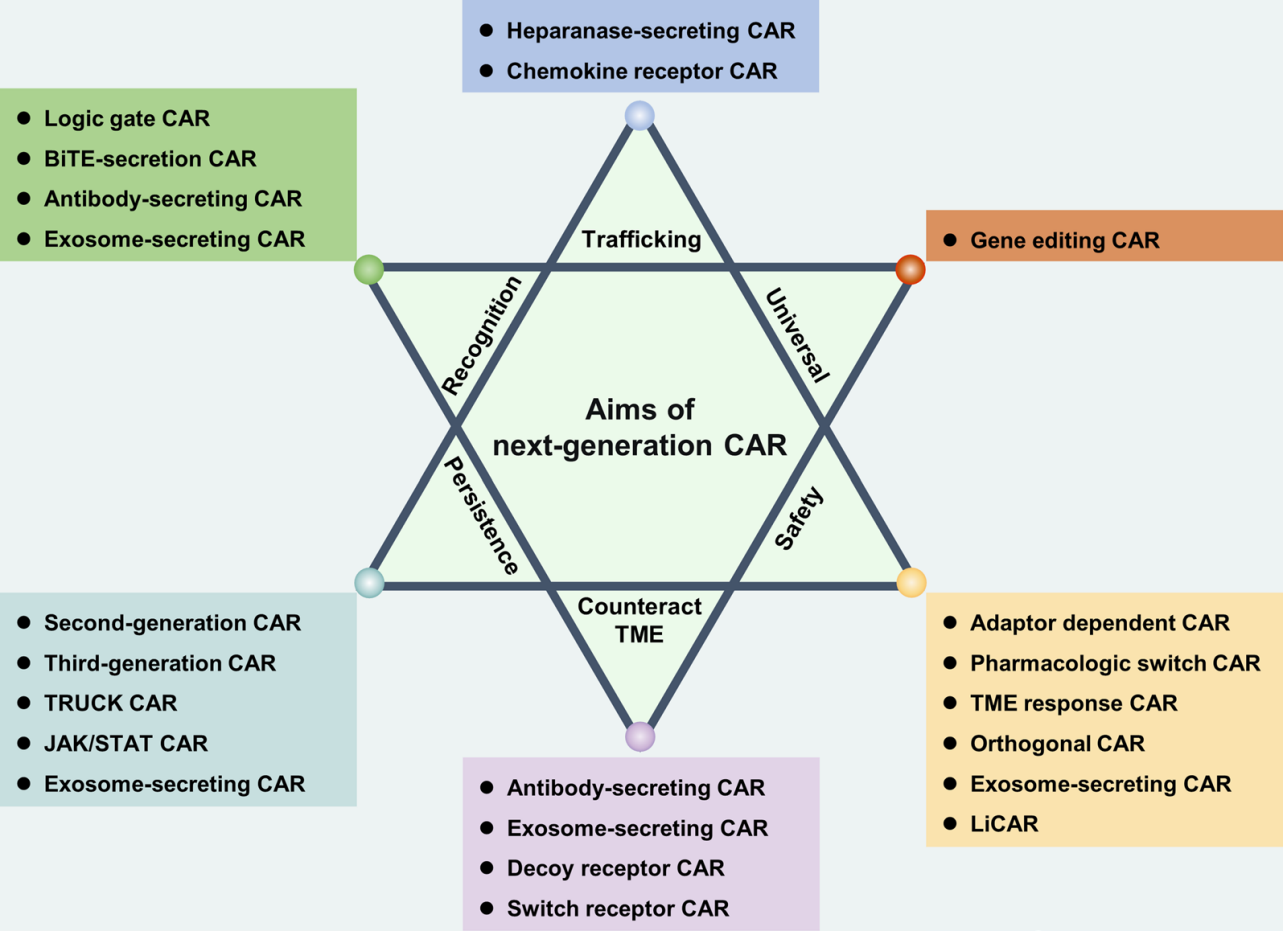
Aims of next-generation CARs and the associated CAR design in the treatment of solid tumors

Current CAR-T cells remain incapable of treating solid tumors. The following issues need to be resolved to further improve CAR-T cell performance: (1) Trafficking: how to localize and enter the tumor tissue; (2) recognition: how to accurately identify tumor cells without harming normal cells; (3) persistence: how to maintain long-term proliferation and anti-tumor ability in vivo; (4) TME resistance: how to resist the inhibition of TME; (5) safety: how to reduce the occurrence of CRS, neurotoxicity (NT), and other side effects; and (6) universality: make CAR-T an off-the-shelf product (Fig. 4). To meet these requirements, researchers have proposed a number of new CAR designs based on the theory of synthetic biology, which we collectively refer to as “next-generation CAR.”

4.1 Logic gates

Based on the theory of engineering, researchers have introduced a series of “circuits” to conventional CAR, termed logic-gate CAR. The activation of logic-gate CAR-T cell therapy relies on a comprehensive signal mediated by multiple activating/inhibitory antigens, which is beneficial to improve the accurate recognition and overcome the heterogeneity of tumor cells. Currently, the logic gates used in CAR-T cell therapy mainly include “OR”, “AND”, and “AND-NOT” loops (Fig. 3Ea–Ec). Each logic gate has a different efficacy and can be modified depending on the actual situation.

1. “OR” logic gate

The simplest method for achieving the “OR” logic gate is via infusion of different CAR-T cell products targeting multiple antigens (cocktail treatment). For example, in clinical trials, CAR-T cells targeting EGFR and CD133 together prolonged the survival time of patients with advanced cholangiocarcinoma [51], and the combination of anti-CD19 and anti-CD20 CAR-T cells also improved the survival time of patients with relapsed B cell acute lymphoblastic leukemia [52]. However, generating multiple types of CAR-T cells increase the workload for researchers and requires more extensive investigation into potential toxicities. Another approach to creating an “OR” logic gate is to use a tandem CAR, which has more than two scFvs or other antigen-recognition motifs in the extracellular domain and can be activated by either of the TAAs. Tandem CAR-T cells targeting CD19/CD20 [53] and CD19/CD22 [54] showed good clinical efficacy in patients with B cell malignancies. Furthermore, CD19/CD20/CD22-targeting CAR-T cells (duoCAR) have shown therapeutic potential for antigen-heterogeneous B cell tumors [55, 56]. However, with the increase in target TAAs, safety needs to be carefully considered. Wong et al. developed a split, universal, and programmable (SUPRA) CAR system that enabled multiple logical controls in CAR-T cells. In the SUPRA CAR system, the CAR is split into two parts: zipCAR and zipFv. zipCAR is located on the cell membrane, and its extracellular domain is a leucine zipper. zipFv is a free scFv fused with a homologous leucine zipper and thus can bind complementarily to zipCAR to form a complete CAR. The SUPRA CAR system can easily switch CAR-T cells to different logic gates using different zipCAR/zipFv pairs. For instance, adding two zipFvs simultaneously to the SUPRA CAR system can rapidly create an OR logic gate, allowing CAR-T cells to potentially target multiple antigens [57]. However, the short half-life of zipFv in vivo limits the application of the SUPRA CAR system.

(2) “AND” logic gate

Few solid tumors can be recognized by a single antigen, and multiple TAAs in combination can more accurately distinguish tumor cells from normal cells. The “AND” logic gate enables CAR-T cells to function only when two TAAs are recognized, which is beneficial for avoiding damage to normal tissues. In the split CAR, the signal domain (CD3ζ) and costimulatory domain (4-1BB or CD28) are separated into two receptors that each recognize a distinct TAA. CAR-T cells obtain complete activation signals only when both TAAs are recognized [58–60]. In a mouse model of prostate cancer, split CAR-T cells targeting prostate-specific membrane antigen (PSMA) and prostate stem cell antigen (PSCA) only attacked target cells expressing both TAAs [58]. However, it must be noted that the CD3ζ-bearing receptor in this design is equivalent to a first-generation CAR, and the binding of this receptor to the antigen alone may cause leaky activation and accelerated exhaustion of CAR-T cells.

Lim et al. developed a novel “AND” logic gate to avoid leaked activation of split CARs. Inspired by the unique function of Notch receptors, they constructed a constitutively expressed synthetic Notch receptor (synNotch) to recognize antigen A, whose activation can release transcription factors to initiate the expression of CAR targeting antigen B. The CAR-T cells enter killing mode when antigen B is recognized [61–65]. In a mouse model inoculated with single-antigen tumors on the left side and double-antigen tumors on the right side, synNotch CAR-T cells only killed double-antigen expressing tumors, but had no effect on tumor cells expressing a single antigen [61]. Although this system can identify tumor cells more accurately, there is an approximately 24 h interval from synNotch activation to CAR activation, which may limit its application [61]. Lim et al. also demonstrated that synNotch CAR-T cells activated by antigen A-positive target cells in the brain of mice had no effect on antigen B–positive target cells inoculated in the abdomen, indicating that synNotch CAR-T cell killing activity was spatially limited [64]. However, for hematological tumors or tumors that metastasized in the blood, the safety of the synNotch system needs to be further explored.

The SUPRA CAR system can also implement “AND” logic control. Using two sets of leucine zips, RR zipCAR (containing RR leucine zipper) and FOS zipCAR (containing human FOS derived zipper domains) were equipped with the costimulatory domain and CD3ζ, respectively. Anti-human epidermal growth factor receptor 2 (HER2) zipFv bound to FOS zipCAR, and anti-Axl zipFv bound to RR zipCAR to form a split CAR. Changing the concentration of each zipFv regulated the intensity of signal output from each receptor [57].

Although the above “AND” logic gates have considerably improved the recognition of tumor cells, leakage still occurs because after the CAR-T cells are activated by antigen A, any cell expressing antigen B can be attacked. Recently, Majzner et al. developed a straightforward, instantaneous, reversible, and leak-free “AND” logic gate based on the current understanding of T cell signaling network. They found that a similar function could be achieved by replacing the intracellular 4-1BB/CD3ζ of the CAR with ZAP-70, which requires linker for activation of T cells (LAT) and SLP-76 for downstream signaling. Therefore, they replaced the two intracellular domains of CAR that recognize both antigens with LAT and SLP-76. After optimization, the generated logic-gated intracellular network (LINK) CAR completely avoided on-target/off-tumor toxicity, and only killed double-positive target cells regardless of the presence or absence of single-positive cells. This showed that the LINK CAR system is safer than synNotch CAR and SUPRA CAR in terms of maintaining an on-target killing effect [66].

(3) “AND-NOT” logic gate

Damage to normal tissues may also be avoided by delivering inhibitory signals with antigens expressed on non-tumor cells. The “AND-NOT” logic gate enables the presence of both activation CAR and inhibition CAR (iCAR) on the surface of CAR-T cells. iCAR typically fuses scFv recognizing a non-tumor antigen to the intracellular domain of an immune checkpoint molecule such as CTLA-4 or PD-1. When iCAR recognizes the antigen, it inhibits the activation of CAR-T cells. In a preclinical study, the activation CAR recognized CD19 and the iCAR recognized PSMA; iCAR-T cells did not kill the target cells expressing both CD19 and PSMA, and the inhibitory effect of iCAR was reversible [67]. The SUPRA CAR system can implement the “AND-NOT” logic gate by using competitive zipFvs. In one example, anti-Axl zipFv competitively blocked the binding of anti-Her2 zipFv to zipCAR, thereby weakening the CAR-T cell attack on Axl^+^/Her2^+^ cells [57].

4.2 Adaptor-dependent

This class of CARs relies on the addition of exogenous ligands and can control the recognition of single or multiple TAAs (Fig. 3Ed). For example, avidin is linked to an extracellular segment of CAR (named biotin-binding immune receptor, BBIR) that can only function when biotinylated antibodies targeting tumor cells are present, and different biotinylated antibodies can be used to target multiple antigens [68, 69]. Similarly, combining anti-fluorescein isothiocyanate (FITC) CAR and FITC-conjugated TAA-targeting antibodies endows CAR-T cells with the function of multi-target recognition [70–73]. In one study, fragment crystallizable gamma receptor (FcγR) was substituted for CAR to confer an antibody-dependent cellular cytotoxicity (ADCC)-like function to engineered T cells. The advantage of this design was the ability to use a clinically approved therapeutic TAA as an adapter [74]. In the SUPRA CAR system, the leucine zipper motif is used to match zipCAR to free zipFv, which also enables CAR-T cells to be activated only in the presence of zipFv. Multiple antigens can be targeted by adding different zipFvs [57].

4.3 Pharmacologic switch

CAR-T cells in vivo may cause fatal side effects if they are out of control, and adverse events such as CRS and NT occur frequently in clinical treatment. To make CAR-T cells a controlled product, researchers have introduced several pharmacologic switch elements (Fig. 3Ee).

1. On-switch

Some CARs function only when a drug is administered. In the Tet-ON system, doxycycline acts as a switch mediator, and reverse tetracycline transcriptional activator (rtTA) can induce CAR expression only in the presence of doxycycline. CAR-T cells targeting CD147 [75], CD19 [76], or CD38 [77] developed based on the Tet-ON system have shown promising anti-tumor effects in vitro or in mouse models. However, because the regulation of CAR by this system occurs at the mRNA level, the control is hysteretic. Bian et al. demonstrated that the expression level of CAR regulated by the Tet-ON system reached the highest level 24 h after doxycycline administration and returned to the baseline value within 48 h after doxycycline removal [75], indicating that this system is not suitable for the situation of CAR-related acute fatal side effects. Additionally, it is necessary to monitor the possibility of antibiotic resistance caused by doxycycline treatment in the clinic. Another method of pharmacological control focuses on costimulatory signals. The small molecule drug rimiducid can dimerize the inducible MyD88/CD40 (iMC) and activate NF-κB to transmit costimulatory signals. In the absence of rimiducid, such CAR-T cells only exhibit first-generation CAR functions and cannot be fully activated. However, in the presence of rimiducid, iMC CAR-T cells show stronger anti-tumor effects than the traditional second- and third-generation CAR treatments [78].

(2) Off-switch

Another strategy to control CAR-T is to induce drugs to turn off CAR signaling or induce CAR-T cell suicide when danger occurs. Duchateau et al. proposed a small molecule protease-based regulation strategy (SWIFF CAR) by sequentially fusing a protease target site, a protease, and a protein degradation component (degron) behind the CAR molecule. Under normal conditions, the protease target site was cleaved and the intact CAR was released. When the exogenous small molecule protease inhibitor asunaprevir was administered, the degron-linked CAR was reversibly degraded. However, this system is also hysteretic, and it can reduce the expression of CAR but cannot completely eliminate its function [79]. Hudecek et al. found that dasatinib, a tyrosine kinase inhibitor clinically approved for the treatment of Philadelphia chromosome-positive chronic myelogenous and acute lymphoblastic leukemias inhibits CD3ζ signaling by interfering with lymphocyte-specific protein tyrosine kinase. In a mouse model, dasatinib rapidly and reversibly prevented CAR-T cell activation and alleviated CAR-T induced CRS, suggesting that dasatinib may be used as an emergency drug to prevent fatal CRS in CAR-T cell treatment [80]. Furthermore, researchers have added a rimiducid-activatable caspase-9 fusion protein to the CAR (iCasp9 CAR) and shown that the activation of caspase-9 leads to CAR-T cell apoptosis. The safety of iCasp9 CAR-T cells has been demonstrated in clinical trials [81]. For patients with graft versus host disease (GvHD) and CRS after receiving allogeneic CAR-T cells, 90% of iCasp9 CAR-T cells were eliminated within 30 min of rimiducid administration, thus preventing the potentially fatal risk [81, 82]. Another approach to deplete CAR-T cells is to force them to express elimination markers such as CD20 and truncated EGFR (loss of intracellular domain). CAR-T cells can be labeled with rituximab (targeting CD20) [83–85] or cetuximab (targeting truncated EGFR) [86], resulting in CAR-T cell elimination by ADCC and complement-dependent cytotoxicity pathways. This approach is advantageous in that clinically approved therapeutic antibodies can be used; however, the slow clearance of CAR-T cells precludes its application in emergency situations.

4.4 Secretion

Arming CAR-T cells to secrete functional molecules that enhance persistence, specificity, and infiltration may further improve their effects on solid tumors (Fig. 3Ef).

Bispecific T cell engagers (BiTEs) consist of two reverse linked scFvs, one targeting TAA and the other typically targeting CD3ζ, that mediate the attachment of T and tumor cells. BiTEs can effectively mobilize bystander T cells and avoid antigen escape. In a mouse leukemia model, anti-CD19 CAR-T cells that secreted CD3/CD19 BiTE exhibited enhanced anti-tumor activity due to mobilization of bystander T cells [87]. In the neuroblastoma, EGFRvIII is a highly specific tumor neoantigen, whereas EGFR is a TAA that is highly expressed in both tumor cells and many normal tissues. EGFRvIII-targeting CAR-T cells caused EGFRvIII-negative escape. However, EGFRvIII-targeting CAR-T cells that secreted CD3/EGFR BiTE lacked this defect. EGFRvIII provided a homing signal for CAR-T cells, and the activated CAR-T cells secreted CD3/EGFR BiTE and mobilized bystander T cells against tumor cells with high EGFR expression [88]. Furthermore, CD3/Fn14 BiTE [89], CD3/B7-H3 BiTE [90], CD3/EGFR BiTE, and CD3/IL13Rα2 BiTE [91] have also shown promising results in preclinical studies of glioblastoma and hepatocellular carcinoma (HCC). However, although CD3/CD19 BiTE blinatumomab is currently approved by the FDA for the treatment of acute lymphoblastic leukemia [92], clinical trials have shown that serious side effects may occur [93]. Therefore, the combination of BiTEs and CAR-T cells should be carefully considered for safety.

Many cytokines can promote T cell proliferation, maintain T cell stemness, and improve the TME. To overcome antigen escape and inhibitory signals in the TME, CAR-T cells can be engineered to secrete activity-enhancing cytokines, and may also induce an endogenous anti-tumor response by remodeling the TME. Researchers have explored a number of cytokines to arm CAR-T cells, known as T cells redirected for universal cytokine-mediated killing (TRUCK). For example, Brentjens et al. showed that CAR-T cells continuously expressing IL-12 exhibit strong proliferation and PD-L1 inhibitory signal resistance in mouse ovarian cancer models, mediate the depletion of tumor-associated macrophages, and significantly prolong the survival time of tumor-bearing mice [94, 95]. This IL-12-secreting CAR-T is currently undergoing clinical trials (NCT02498912) [96]. Moreover, CAR-T cells secreting IL-7 [97], IL-12 [98–101], IL-15 [102–107], IL-18 [108–110], IL-33 [111], and IL-36γ [112] have also exhibited improved persistence or anti-tumor ability in preclinical or clinical trials; however, the continuous expression of pro-inflammatory cytokines such as IL-12 may lead to systemic toxicity [113, 114]. In contrast, the synNotch system developed by Lim et al. achieved regulation of cytokine release and has exhibited ideal results [115]. Xu et al. found that the autocrine IL-23 signal also enhanced the persistence and anti-tumor ability of CAR-T, with fewer side effects than CAR-T cells secreting IL-15 or IL-18 [116].

The extracellular matrix (ECM) of tumor tissue is one of the main factors limiting the infiltration of CAR-T cells. Secreted ECM-degrading enzymes can enhance CAR-T cell infiltration in solid tumors. Dotti et al. modified CAR-T cells to express heparanase, which can degrade heparan sulfate proteoglycans, the main components of the ECM. Anti-disialoganglioside (GD2) CAR-T cells expressing heparanase showed enhanced infiltration in solid tumors and were associated with significantly prolonged survival time in mice [117]. However, in a clinical trial, treatment with the pegylated form of ECM-degrading enzyme hyaluronidase increased the occurrence of thromboembolic events and reduced the overall survival of patients with metastatic pancreatic cancer [118], suggesting that ECM-degrading enzyme-expressing CAR-T cells may cause fatal side effects.

CAR-T cells have also been engineered to secrete antibodies targeting PD-L1 to enhance the clearance of PD-L1-expressing tumors through ADCC [119]. Similarly, the secretion of anti-PD-1 scFv by CAR-T cells can block the inhibitory signal of the TME and enhance the anti-tumor effect [120].

Furthermore, the interaction of herpes virus entry mediator (HVEM) and B and T lymphocyte attenuator (BTLA) generates inhibitory signals. Although the loss of HVEM leads to B cell proliferation and the development of germinal center lymphoma, CAR-T cells can deliver the extracellular portion of HVEM to the tumor site to restore inhibitory BTLA signaling and kill lymphoma cells. This shows that CAR-T can also function as drug delivery vectors [121].

CAR-T can also act as a drug delivery vector by secreting exosomes. Exosomes have many biological functions as mediators of intercellular communication and molecular transfer. Owing to their small size, exosomes can effectively cross the barrier of solid tumors and have shown potential in the treatment of solid tumors. Minn et al. engineered CAR-T cells to secrete exosomes carrying the stimulatory RNA RN7SL1 and reported promising therapeutic effects in solid tumor models [122]. RN7SL1 has many functions and was found to promote expansion and effector memory differentiation and enhance the function and persistence of CAR-T cells. Moreover, exosomes containing RN7SL1 selectively transferred to immune cells, restricted myeloid-derived suppressor cell (MDSC) development, reduced the expression of inhibitory cytokine TGF-β in myeloid cells, and promoted the costimulatory phenotype of dendritic cells (DCs). Moreover, equipping these exosomes with peptide antigens can mobilize endogenous immune cells to attack tumor cells with CAR antigen loss [122]. Therefore, multi-armed CAR-T cells secreting RN7SL1-carrying exosomes can effectively infiltrate tumors, improve the TME, mobilize bystander immune cells and provide them with antigens, and significantly improve the efficacy of anti-solid tumors [122]. Furthermore, exosomes secreted by CAR-T cells have many advantages, such as containing surface CAR but not PD-1 and containing perforin and granzyme B without the risk of CRS. Therefore, these exosomes can target and clear tumor cells similarly to parental T cells but are not limited by TME, and they exhibit good safety. This makes purified exosomes derived from CAR-T a promising tool for the treatment of solid tumors [123, 124].

4.5 TME response

In addition to immunosuppressive cells and cytokines, the TME is characterized by several other abnormal physical factors, such as acidity (pH 6.0–6.9) [125] and hypoxia (O_2_ < 2%) [126], which also limit the anti-tumor effect of immune cells [127–129]. However, new functions of CAR-T can be developed by taking advantage of these abnormal physical factors. TME response CAR can limit the activation and distribution of CAR-T cells to limit systemic on-target/off-tumor toxicity (Fig. 3Eg). For example, hypoxia-inducible factor 1α (HIF-1α) contains an oxygen-dependent degradation domain (ODD), and fusing the ODD to the CAR molecule can mediate CAR degradation under normoxic conditions. Consequently, the CAR is only expressed in the hypoxic TME, reducing the damage to many normal tissues [130, 131]. Based on the oxygen-concentration-sensing characteristic of HIF-1α [132], Xu et al. introduced ODD into CAR and added hypoxia response elements (HREs) before the gene encoding CAR, achieving hypoxia-induced high expression and normoxia-mediated degradation of CAR [133]. CAR-T cells that sense an acidic environment are also in development. After screening a series of scFvs targeting HER2, Frost et al. obtained one with the best recognition activity in an acidic environment and constructed a pH-sensitive CAR-T that functioned only in an acidic TME, resulting in the regression of HER2-positive tumors in a mouse model [134]. However, it should be noted that some non-malignant tissues may share the physical characteristics of the TME, such as the physiologically hypoxic renal medulla and intestinal mucosa [135, 136] and the acidic gastric mucosa. Therefore, TME-responsive CARs also need to respond to highly specific TAAs or act in combination with other designs that improve specificity to avoid attacking normal tissues.

4.6 Other membrane protein modifications

In addition to the above designs, many other membrane protein modification schemes have been developed (Fig. 3Eh). For instance, IL-2 is generally used to stimulate the survival and expansion of CAR-T cells in vitro, but the administration of natural IL-2 in vivo can also cause indiscriminate activation of other immune cells such as NK and T cells, which may cause unpredictable side effects [137, 138]. Therefore, researchers have modified the IL-2/IL-2Rβ pair to design an orthogonal IL-2/IL-2Rβ system [139, 140]. The modified IL-2 can only activate CAR-T cells expressing the modified IL-2Rβ and do not react to native IL-2Rβ, thus avoiding non-specific activation. Moreover, the modified IL-2Rβ can only be activated by the modified IL-2 but has no response to natural IL-2, enabling precise regulation of orthogonal CAR-T cells in vivo. CAR-T with an orthogonal IL-2/IL-2Rβ system has been demonstrated to achieve complete response in a mouse refractory lymphoma model, and the number of CAR-T cells can be controlled to avoid CRS [139, 140]. Hirano et al. introduced IL-2Rβ and STAT3 binding motif (YXXQ) to the intracellular domain of a second-generation CAR. Owing to the addition of cytokine-mediated activation signals (JAK/STAT), this novel CAR-T cell showed better proliferation and anti-tumor activity than the unmodified second-generation CAR and prevented terminal differentiation in mice [141].

As tumor cells can secrete chemokines, the corresponding chemokine receptors can be used to enhance the homing of CAR-T cells to tumor tissues. For example, human HCC tumor tissues and cell lines express high levels of CXCR2 ligands, whereas T cells lack CXCR2. Therefore, Lin et al. introduced CXCR2 into CAR-T cells and found that they significantly enhanced their infiltration in HCC tissues relative to that of CAR-T without CXCR2 [142]. Furthermore, studies have shown that NSCLC tumor tissues highly express the chemokine MCP-1, while its receptors CCR2b and CCR4 are expressed at low levels on activated T cells. Zhu et al. constructed CAR-T cells expressing CCR2b to enhance the infiltration and anti-tumor function of CAR-T cells [143]. Similarly, CAR-T cells carrying colony-stimulating factor (CSF)-1R [144], CCR4 [145], and CCR2b [146, 147] have also shown promising results in various solid tumor models in preclinical studies. However, this requires high specificity of the chemokine, and more studies are needed to ensure that the chemokine receptor system will not cause CAR-T cells to target normal tissues.

Researchers have constructed a series of modified membrane receptors to block inhibitory signals for CAR-T cells. For example, CAR-T cells are forced to express PD-1 [148] or TGF-β receptors [149] that lack intracellular signaling domains (dominant-negative receptors). These receptors can competitively bind to PD-L1 or TGF-β but do not transmit inhibitory signals, thus enhancing the persistence of CAR-T cells and their ability to resist the TME. CAR-T cells expressing modified dominant-negative Fas blocked FasL-mediated apoptosis in the TME and also improved resistance to the TME [150].

Furthermore, modified membrane proteins can also convert inhibitory signals to activating signals (switch receptor). For example, the extracellular and intracellular domains of PD-1 and CD28, respectively, can be fused to transmit activation signals when stimulated by PD-L1 [151]. In clinical trials, anti-CD19 CAR-T expressing a PD-1/CD28 switch receptor was administered to patients with R/R B cell lymphoma, resulting in an overall response rate of 58.8% and a CR rate of 41.2% [152]. Similarly, switch receptors that fuse the extracellular domain of inhibitory IL-4R with activating IL-7R [153, 154], IL-2Rβ [155], or IL-21R [156] have also been shown to maintain the pro-inflammatory phenotype of CAR-T cells while exhibiting good TME resistance. However, it should be noted that excessive activation of CAR-T cells may cause side effects such as CRS and NT.

CD40 can be expressed in antigen-presenting cells (APCs), fibroblasts, endothelial cells, and certain hematopoietic and epithelial tumor cells. CAR-T cells expressing CD40 ligand (CD40L) can not only directly enhance the killing of CD40-positive tumor cells but also activate APCs and mobilize other endogenous immune cells to participate in anti-tumor responses and avoid tumor immune escape [157, 158].

Another promising design to enhance the temporal and spatial controllability of CAR-T is the regulation of CAR-T cells using light. Zhou et al. separated the intracellular signal domains of traditional CAR and fused them with optical dimer sensing elements so that they could be assembled into a complete CAR only when exposed to blue light. After extensive screening, they found that light-oxygen-voltage domain 2 (LOV2)-based optical dimers could mediate optimal light regulation, and therefore designed light-switchable CAR (LiCAR). In subsequent cytotoxic assays, LiCAR-T cells lysed target cells only in the presence of both tumor antigen and blue light. However, the ability of blue light to penetrate tissues is poor. To improve the clinical applicability of this technology, the researchers integrated upconversion nanoplates (UCNPs), which are injectable nanoparticles that emit blue light upon exposure to near infrared light (with strong tissue penetration ability). Therefore, LiCAR-T cells co-infused with UCNPs showed significant and controllable tumor killing in vivo, greatly improving the safety of CAR-T cell therapy [159]. Furthermore, certain other attempts based on light-regulated CAR-T have been made, all of which have shown promising safety [160–162].

4.7 Gene editing

Gene editing technology has been used to develop new CAR-T cells (Fig. 3Ei). Knockout or silencing of *PDCD1* by CRISPR/Cas9 [163, 164] or short hairpin RNA (shRNA) [148] has been shown to increase anti-tumor effects. In one clinical trial, all 20 patients with NSCLC who received PD-1 knockout anti-MUC1 CAR-T cells experienced significant symptom improvement, and 11 of them achieved stable disease status [165]. However, some studies have shown that PD-1 silencing may not be conducive to the anti-tumor effect of CAR-T cells. Han et al. found that a blockade of PD-1 limited the proliferation and promoted the early differentiation of CAR-T cells [166], suggesting that further research is needed to investigate how this approach affects CAR-T function. Knockdown of CTLA-4 with shRNA also significantly increased proliferation and anti-tumor activity in first- but not second-generation CAR-T cells [167].

The T cells used for CAR-T cell therapy are often derived from the patient to avoid allogeneic immune rejection. Therefore, individualized CAR-T cells must be temporarily generated for each patient, which has limitations such as high cost, long waiting time, and inconsistent CAR-T cell quality. Another application of gene editing technology in CAR-T cell therapy is to create off-the-shelf products also known as universal CAR-T cells. The generation of universal CAR-T cells needs to solve two major problems: graft versus host disease (GvHD) and host versus graft rejection (HvGR). This can be achieved by using gene editing techniques (zinc finger nucleases [ZFNs], TALENs, and CRISPR/Cas9) to knock out TCR and MHC-I or CD52 on allogeneic CAR-T cells [168–171]. TCR site destruction and CAR coding gene insertion by CRISPR/Cas9 has been reported to result in potentially universal CAR-T cells that effectively reduced tonic signaling and delayed exhaustion [172, 173]. Anti-CD19 CAR-T cells modified with TCRα constant chain knockout by TALENs have been shown in clinical trials to be effective and safe for patients with relapsed or refractory B cell acute lymphoblastic leukemia [174, 175]. The Cas9 variants developed by Ciaramella et al. achieved simultaneous knockout of MHC-I, MHC-II, and TCR in T cells and may find application in the development of universal CAR-T cells [176]. However, universal CAR-T cell therapy continues to face considerable technical obstacles in clinical application, including low editing efficiency and off-target editing. In contrast, replacing T cells with other MHC-independent immune cells (such as NK cells) may make universal cell therapy more feasible.

#### Research progress

Emily Whitehead, then a 6-year-old with acute lymphoblastic leukemia, was treated with Kymriah (anti-CD19 CAR-T cells) in 2012 and has been free of the disease for 11 years, making her the first patient with leukemia to be cured by CAR-T therapy. This success caused CAR-T cell therapy to receive considerable attention and undergo rapid development. CAR-T cell immunotherapy has achieved great success in the treatment of hematologic malignancies. Eight CAR-T cell immunotherapies have been approved for marketing (Table 1), of which Kymriah, Yescarta, Tecartus, Breyanzi, Abecma, and Carvykti are FDA approved for the treatment of relapsed or refractory (r/r) B cell precursor acute lymphoblastic leukemia, r/r large B cell lymphoma, r/r mantle cell lymphoma, r/r multiple myeloma, and r/r follicular lymphoma. Carteyva and CT103A are approved by the China National Medical Products Administration for the treatment of r/r large B cell lymphoma and r/r multiple myeloma, respectively (Table 1).

Furthermore, some CAR-T therapies that remain in clinical trials have also shown encouraging outcomes. For example, Arcellx Inc. announced the phase 1 clinical data of their B cell maturation antigen (BCMA)-targeted CAR-T therapy for the treatment of multiple myeloma (NCT04155749) in April 2022. The ORR was as high as 100%, and the CR rate was 75% [177]. In phase 2 clinical results of KTE-X19, a CD19-targeted CAR-T cell therapy developed by Kite Pharma showed an 85% ORR and 59% CR rate in 74 patients with r/r mantle cell lymphoma (NCT02601313) [178]. Huang et al. specifically inserted CAR into the *PDCD-1* locus by electroporation to produce non-viral anti-CD19 CAR-T cells that led to a complete response in seven out of eight patients (87.5%) without significant side effects (NCT04213469) [179]. These results have set high expectations for novel CAR-T therapies.

However, it is important to note that these CAR-T therapies with promising clinical outcomes are mainly employed to treat hematological tumors. CAR-T therapies for solid tumors are being developed rather slowly due to the limitations of target specificity and the immunosuppressive TME. Currently, mesothelin, glypican-3, GD2, HER2, B7-H3, and claudin18.2 are the main targets of CAR-T cells for the treatment of solid tumors (Fig. 2C, Additional file 1), including glioma and colorectal, cervical, pancreatic, and lung cancers. However, these CAR-T therapies are all in phase 1 or phase 2 clinical trials, and published data show that the response rate of CAR-T cell therapy for solid tumors is weaker than that for hematological tumors. In one study, for example, all 37 patients with gastrointestinal cancer experienced grade 3 or higher hematologic toxicity after receiving various doses of claudin18.2-targeted CAR-T cell therapy, and 94.6% of patients experienced grade 1 or 2 CRS with an ORR of 48.6% (NCT03874897) [180]. In a clinical trial of anti-EGFRvIII CAR-T cell therapy for recurrent glioblastoma, 0 out of 10 patients achieved partial or complete response (NCT02209376) [181], despite good efficacy against EGFRvIII positive tumor cells in vitro and in xenogeneic mouse models [182, 183]. Therefore, CAR-T cell treatments for solid tumors are still a long way from being used in clinical settings.

Furthermore, as CAR-T cell therapy develops, its potential in the treatment of other diseases is gradually becoming apparent. For example, CAR-T cell therapy targeting HIV surface proteins almost eliminated HIV in humanized mice [184], and anti-gp120 CAR-T therapy is being tested in patients with HIV (NCT04648046). CAR-modified Treg cells can exert specific immunosuppressive functions and avoid GvHD after organ transplantation [185]. QEL-001, an HLA-A2 targeted CAR-Treg cell therapy developed by Quell Therapeutics, has entered clinic trials (NCT05234190) for the prevention of GvHD after liver transplantation [186]. Additionally, the potential of CAR-Treg cell therapy for the treatment of autoimmune diseases, particularly systemic lupus erythematosus, has been highlighted and clinical trials have been initiated in recent years [187, 188].

#### Challenges and potential solutions

Current CAR-T therapy has many challenges that need to be overcome, particularly with regard to the treatment of solid tumors (Fig. 1), where it has not yet achieved breakthrough success.

1. Target selection

A suitable target is the first consideration for CAR-T cell therapy. Although both CAR-T and TCR-T cells mediate tumor killing by loading T cells with a receptor that specifically recognizes tumor antigens, the structures of these two receptors are different. Consequently, CAR does not share some of the advantages of TCR, which restricts the range of selectable targets. First, fewer targets are available for CAR-T cells than for TCR-T cells because CAR is MHC-independent and can only detect surface antigens on the plasma membrane (only around 25% of human proteins are membrane-bound) [189]. Second, compared to TCR, CAR has substantially lower antigen sensitivity than TCR. TCR can be activated by 1–50 MHC molecules, whereas CAR requires at least 1000 antigens [13], meaning that low antigen density is insufficient for CAR-T therapy.

The ideal target is confined to tumor cells and plays a critical role in their growth. However, most CAR-T therapy targets are also expressed in normal tissues and thus contribute to the on-target/off-tumor toxicity of CAR-T cell therapy. The severity of this toxicity is related to the expression and importance of the selected target in normal tissues. CD19 is now the most developed target in CAR-T cell therapy (Fig. 2C, Additional file 1). As CD19 is a marker of human B cells, healthy B cells are also eliminated by anti-CD19 CAR-T cell therapy, drastically lowering serum immunoglobulin levels. However, the human body can withstand the loss of B cells over a short time period, paving the way for the widespread application of CD19 in the field of CAR-T cell treatment for B cell lymphoma [190]. However, screening for highly specific targets for solid tumors is challenging due to the considerable heterogeneity. Although a growing number of target studies have been conducted recently, many of which have progressed to clinical trials (Fig. 2A, C), all of the CAR-T cell therapies under investigation exhibit varying degrees of target-related side effects. For example, multiple HER2-targeting CAR-T therapies are being investigated for efficacy and safety in HER2-positive tumors. However, in a trial of HER2-targeted CAR-T cell therapy for colon cancer with lung and liver metastases, a patient receiving CAR-T cells developed rapid respiratory failure, lung invasion, and multiple organ dysfunction, ultimately leading to death [191]. CAR-T cells may recognize HER2 expressed in non-malignant tissues, thereby triggering systemic CRS and the destruction of normal organs [191]. Furthermore, carbonic anhydrase IX (CAIX)-targeted CAR-T cell therapy in renal cell carcinoma trials resulted in liver enzyme abnormalities due to CAR-T cell infiltration in the bile duct epithelium expressing CAIX. However, the use of anti-CAIX monoclonal antibodies can prevent these side effects, providing evidence for off-tumor toxicity of CAR-T cell therapy [192, 193]. Alternatively, CAR-T cells targeting carcinoembryonic antigen (CEA) have also been linked to severe colitis when used to treat colon cancer [194].

Next-generation CAR-T cells with logic-gate control can partially overcome the constraints of antigen heterogeneity and specificity. For instance, the “OR” logic-gate CAR, which mainly includes tandem and dual CARs, can recognize two or even three antigens and thus more thoroughly removes heterogeneous tumor cells. In terms of improving specificity, the design of “AND” logic-gate CAR, such as the split and synNotch CARs, ensures that cytotoxicity only occurs when two antigens are recognized (Fig. 3). Another effective strategy for tackling the target issue is to combine oncolytic viruses (OVs) with CAR-T cell therapy. OVs carrying specific genes can selectively infect tumor cells and replicate or express selected genes within them. OVs can be used to stimulate tumor cells to express tumor antigens of interest, which can then be targeted by CAR-T cells to further eliminate tumor cells. In 2020, Priceman et al. reported that the use of anti-CD19 CAR-T cells in combination with OV19t (an OV that induces cancer cells to express a truncated non-signaling variant of CD19) resulted in a complete response in approximately 60% of mice as opposed to 22% of mice treated with OV19t alone [195]. Finally, employing library approaches for multiple targeting can also address the restrictions of targets. The adaptive immune system relies on a large and diverse repertoire of antibodies for antigen recognition. Recently, researchers created an engineered immune cell repertoire that recognizes over 10^6^ potential antigens to mimic this mechanism in immunotherapy. They discovered that this library could recognize non-self-antigens and exhibit antigen-dependent clonal expansion, resulting in an increased population of tumor-specific effector cells and long-lasting anti-tumor responses. Moreover, the synthetic library led to robust immunological memory and the recognition of mutated or evolved tumors owing to the maintenance of CAR diversity [196]. Similar to TIL therapy, this design has advantages in that it can target multiple antigens and thus overcome the issue of heterogeneity. Furthermore, because the antibodies used in library construction have been screened by the human immune system and have appropriate affinity, the artificial library will not be toxic to normal tissues. However, the clinical feasibility of this technology has not yet been established. Using the CAR library from one patient may not have the same effects on other individuals because antigen expression profiles and immunological conditions vary among them. Additionally, it is now unrealistic to customize the CAR library for each patient because the process requires considerable time and effort.

2. Infiltration

CAR-T cells can be widely distributed throughout the blood and lymphatic systems and therefore are likely to encounter cancerous cells in hematological tumors. However, CAR-T cells face challenges with infiltrating solid tumors due to their inherent chemotactic defects and the external restrictions imposed by the dense ECM, which is composed of highly organized fibrous molecules, glycoproteins, and other macromolecules [197, 198]. Contrary to TILs that have received chemotactic training, CAR-T cells generated from peripheral T cells typically exhibit imperfect chemotactic capacity. Chemokine signals can therefore be employed to facilitate the infiltration of CAR-T cells. For instance, forced expression of CSF-1 receptor in CAR-T cells boosts their anti-tumor activity against CSF-1-rich solid tumors [144]. Furthermore, combining CAR-T cells with OVs is another method that may promote infiltration [199, 200]. In a preclinical study, GD2-targeting CAR-T cells were combined with an OV expressing the chemokine CCL5 and the cytokine IL-15. This combination enhanced the infiltration and persistence of CAR-T cells and significantly improved the survival of tumor-bearing mice [201]. Additionally, two recent studies have also demonstrated the feasibility of enhancing CAR-T cell infiltration by combining them with OVs. In a mouse glioblastoma model, the combination of B7-H3-targeted CAR-T cells with oncolytic adenoviruses expressing the chemokine CXCL11 increased the infiltration of CAR-T cells and reprogrammed the immunosuppressive TME [202]. Furthermore, in a mouse renal cell carcinoma model, oncolytic adenoviruses expressing CCL5 and IL-12 enhanced CAR-T cell infiltration and inhibited tumor growth when combined with CAIX-targeting CAR-T cells [203]. Physical barriers created by the ECM are another major hurdle impeding CAR-T penetration. CAR-T cells that secrete ECM-degrading enzymes can successfully dissolve physical barriers, enhancing CAR-T infiltration [117]. Furthermore, local injection can also be used to directly deliver CAR-T cells into the tumor, bypassing the barriers and minimizing on-target/off-tumor effects. This approach has been attempted in brain [204], breast [205], and liver [206] cancers. However, many solid tumors are metastatic or have numerous lesions, limiting the applicability of local injections.

3. Exhaustion

Extensive in vitro expansion, repetitive stimulation by tumor cells, and the inhibitory TME all result in CAR-T cell exhaustion and a loss of anti-tumor function [117, 207]. Exhausted CAR-T cells exhibit upregulated inhibitory receptors (e.g., PD-1, Lag3, Tim3, and TIGIT); decreased secretion of IL-2, TNF-α, and IFN-γ; altered metabolism; and epigenetic modifications.

Many strategies are currently being used to overcome exhaustion in CAR-T cell therapy. In terms of immune checkpoint blockade-based strategies, antibodies targeting PD-1 and PD-L1 have been used in combination with CAR-T cells [148, 208–210]. For example, Brown et al. found that an in vitro PD-1 blockade significantly improved the function of anti-GD2 CAR-T cells after repeated antigen stimulation. They also demonstrated that anti-GD2 CAR-T cells, when combined with anti-PD-1 antibodies, exhibited greater persistence in metastatic melanoma patients [210]. Another clinical trial conducted by Adusumilli et al. yielded similarly encouraging results, with 8 out of 11 patients with mesothelioma responding to anti-mesothelin CAR-T cells and anti-PD-1 antibodies, including complete metabolic responses in two patients [211, 212]. In addition to PD-1, targeting PD-L1 can also prevent the exhaustion of CAR-T cells. Katz et al. found that PD-L1 expression in liver MDSCs inhibited the anti-tumor function of CAR-T cells, while the use of anti-PD-L1 antibody improved the efficacy of CAR-T cells [213]. OVs have also been used to prevent CAR-T cell exhaustion and were shown to enhance the anti-tumor effect of CAR-T cells when equipped with the anti-PD-L1 mini-antibody [214]. In addition to the addition of exogenous antibodies, strategies to generate CAR-T cells with endogenously expressed immune checkpoint blockade antibodies or decoy immune checkpoints are equally feasible [119, 120, 215]. Brentjens et al. developed CAR-T cells secreting anti human PD-1 scFvs that outperformed conventional CAR-T cells in terms of their in vivo anti-tumor effects [120]. To block the PD-L1/PD-L2-mediated inhibitory signals, Adusumilli et al. engineered CAR-T cells to express dominant-negative PD-1 with intracellular domain deletion. They demonstrated that the truncated PD-1 rescued the CAR-T cell function from PD-1 ligand-mediated inhibition both in vitro and in vivo [148]. Furthermore, they discovered that this cell-intrinsic blocking by a dominant-negative receptor can achieve similar effects to an extrinsic blockade via anti-PD-1 antibody administration [216]. Moreover, Moon et al. developed CAR-T cells with a “switch receptor” that fused the extracellular domain of PD-1 with the transmembrane/intracellular domain of CD28. Such switch receptors converted the inhibitory signal of PD-1 into an activation signal and thus improved the persistence and anti-tumor effects of CAR-T cells in mesothelioma and prostate cancer mouse models [151]. In addition to immune checkpoint blockades, direct knockdown or knockout of the immune checkpoints through shRNA or gene editing technologies such as CRISPR/Cas9 or TALEN can also prevent CAR-T cell exhaustion [148, 163, 164, 217, 218]. Wei et al. used CRISPR/Cas9 to knockout PD-1 in mesothelin-targeting CAR-T cells. The PD-1 knockout CAR-T cells exhibited stronger cytokine production and cytotoxicity toward PD-1-positive tumor cells and performed better in terms of tumor control and relapse prevention in vivo than conventional CAR-T cells with or without anti-PD-1 antibody treatment [164]. Zhao et al. used the CRISPR/Cas9 system to simultaneously disrupt TCR, β-2 microglobulin, and PD-1 genes to construct a universal CAR-T cell that showed a stronger anti-tumor effect in vivo [218].

According to the marker and stemness, T cells can be divided into Tn, Tscm, Tcm, effector memory T cells, effector T cells, and terminally differentiated T cells [219]. The therapeutic effect and persistence of CAR-T cells are related to the differentiation state of the cells, as less differentiated T cells can self-renew [220]. Clinical studies have found that the proportion of Tn, Tscm, and Tcm in CAR-T cells is positively correlated with the overall response in various malignant tumors, including melanoma [221, 222], neuroblastoma [223], chronic lymphocytic leukemia [224], multiple myeloma [225, 226], pancreatic cancer [227], and B cell lymphoma [228–231]. These studies demonstrate the importance of a less differentiated status of CAR-T cells.

The function and differentiation of many immune cells, particularly T cells, are closely linked to metabolic state. Generally, less differentiated T cells, which have lower metabolic requirements, use the glucose-derived pyruvate or fatty acid oxidation (FAO) pathway to obtain ATP via oxidative phosphorylation (OxPhos) [232–234]. In contrast, effector T cells mainly utilize glycolysis to provide ATP and intermediates for proliferation and function, particularly after encountering antigens [234, 235]. It has been shown that T cell differentiation can be controlled by glycolysis and OxPhos or FAO pathways [236, 237]. The complex TME can affect T cell metabolism in various ways, including via the consumption of key nutrients (e.g., glucose [238] or tryptophan [239]), accumulation of ions (e.g., potassium [240, 241]) and biologically active metabolites (e.g., lactate [242, 243] or adenosine [244–246]), oxidative stress (e.g., reactive oxygen species (ROS) [247, 248]), and immune checkpoints. Attempts have been made to enhance stemness by reprogramming CAR-T cell metabolism. Studies have found that CD28 costimulatory domain-based CAR-T cells exhibit a higher level of glycolysis, while 4-1BB based CAR-T cells have higher basal oxygen consumption and OxPhos rates, reflecting the metabolic characteristics of memory-like T cells [45]. This is consistent with the better persistence of the 4-1BB-based CAR-T cells. IL-7, IL-15, or IL-21 can be used to replace IL-2 in the preparation of CAR-T cells in vitro, as IL-2 promotes glycolysis and late differentiation of T cells [249]. However, IL-15 can shift energy metabolism from glycolysis to OxPhos by inhibiting mTOR, maintaining stemness and inducing stronger anti-tumor activity and proliferation [250]. The combination of IL-15 and IL-7 has also been shown to induce the Tscm phenotype of CAR-T cells [251], and IL-21 can enhance FAO and promote Tcm formation [252]. Inhibition of mTOR or the upstream Akt can also promote T cell stemness by switching T cell metabolism to the FAO pathway [253–255]. Treating T cells with 2-deoxy-D-glucose to interfere with glycolysis during in vitro expansion is conducive to memory T cell formation [256]. Furthermore, studies have found that interference in the PD-1/PD-L1 pathway can shift T cell metabolism from glycolysis to FAO and OxPhos, thereby promoting cell survival and self-renewal [257]. The combination of anti-PD-1 antibody and the PGC1α agonist bezafibrate can increase OxPhos and reduce T cell apoptosis [258]. ROS accumulation in activated T cells is detrimental to T cell function. Anti-CD19 CAR-T cells supplemented with the antioxidant N-acetylcysteine limit ROS metabolism, reduce glycolysis, promote FAO, and stimulate T cell differentiation into Tscm [259]. Competition for nutrients within the TME also affect T cell phenotypes. Studies have found that L-arginine promotes memory formation and enhances OxPhos in T cells, improving their anti-tumor function [260]. Cholesterol is also critical for T cell function because it affects TCR aggregation and the formation of immune synapses. In a mouse model of melanoma, inhibition of cholesterol esterification led to elevated plasma membrane cholesterol levels and enhanced the function of CD8^+^ T cells [261]. Furthermore, Xu et al. showed that T cells within tumors are cholesterol deficient, which leads to T cell exhaustion and dysfunction. The depletion of LXRβ, a molecule that downregulates cell cholesterol, improved the anti-solid tumor function of CAR-T cells [262]. Short chain fatty acids can also regulate the anti-tumor activity of CAR-T cells through metabolic and epigenetic reprogramming. CAR-T cells treated with pentanoate and butyrate reportedly exhibited increased mTOR activity and reduced class I histone deacetylase (HDAC) activity. This reprogramming enhanced the anti-tumor activity of ROR1-targeting CAR-T cells [263].

In addition to metabolic reprogramming, the persistence of CAR-T cells can also be improved by reprogramming gene expression. Research on CAR-T cells from complete response and non-response patients has found that the gene expression profile of exhausted T cells significantly differs from that of effector and memory T cells [224] and is regulated by epigenetic modifications and transcription factors [264]. In exhausted cells, the expression of epigenetic regulators such as DNA methyltransferase (DNMT) and HDAC are considerably changed. DNA methyltransferase 3A (DNMT3A) has been shown to downregulate the factors responsible for memory cell maintenance and T cell exhaustion [265, 266]. Similarly, methylcytosine dioxygenase TET2 catalyzes DNA methylation to promote T cell exhaustion [264]. It has been shown that the transcriptional profile of TET2-depleted CAR-T cells exhibiting a central memory phenotype showed increased anti-tumor effects [267, 268]. PR domain zinc finger protein 1 (PRDM1) regulates gene expression by interacting with various epigenetic regulatory enzymes [269] and can promote T cell terminal differentiation by negatively regulating memory-related genes [270]. The depletion of PRDM1 in CAR-T cells reshaped chromatin openness in approximately 7000 genomic regions and affected the expression of over 2000 genes. PRDM1-deficient CAR-T cells show a stemness phenotype, and although their effector functions, such as granzyme B and perforin production, are somewhat diminished, their good persistence allows them to exhibit potent anti-tumor effects in vivo [271].

Many transcription factors, such as T-bet, Eomes, NFAT, Blimp-1, BATF, Foxo1, and Foxp1, are associated with T cell exhaustion [109, 272–276]. Complete knockout of Eomes impaired T cell development, whereas deletion of one allele alleviated CD8^+^ T cell exhaustion [277]. T-bet was shown to reduce the expression of inhibitory receptors such as PD-1. Low glucose and hypoxia in the TME downregulate T-bet expression and promote T cell exhaustion [278]. T-bet and Eomes compete for the same binding sites, including *PDCD1*. The subcellular localization of Eomes and T-bet is critical for their regulatory functions during exhaustion. Exhausted T cells have a higher nuclear Eomes/T-bet ratio than memory T cells [279, 280]. TCF-1 is a key transcription factor of progenitor exhausted CD8^+^ T cells that can regulate the balance of T-bet/Eomes transcription factors and drive the fate of CD8^+^ T cells by promoting Eomes expression [281]. Therefore, targeting TCF-1 may promote the persistence of CAR-T cells. IL-18-secreting CAR-T cells constructed by Abken et al. can upregulate T-bet and downregulate FoxO1, showing good effects on advanced solid tumors [109]. The transcription factor NFAT combines with activator protein 1 (AP1) to activate genes involved in T cell activation and upregulate the effector cytokines IL-2 and IL-4 [282]. However, in the absence of AP1, NFAT binds to other promoters and triggers the expression of exhaustion-related genes [283]. TOX and NR4A are downstream transcription factors regulated by NFAT and cooperate to promote CD8^+^ T cell exhaustion [284]. However, triple knockout of *Nr4a* (including *Nr4a1*, *Nr4a2*, and *Nr4a3*) in CAR-T cells promoted tumor regression and prolonged the survival of tumor-bearing mice [285]. June et al. showed that SOX4 and ID3 are the key transcription factors for CAR-T cell exhaustion, and the downregulation of their expression can improve the therapeutic effect on solid tumors by delaying CAR-T cell dysfunction [286]. McCutcheon et al. systematically profiled the effects of activation and repression of 120 transcription factors and epigenetic modifiers on human CD8^+^ T cell state through orthogonal CRISPR screening, and discovered that the basic leucine zipper ATF-like transcription factor 3 (BATF3) promotes memory T cell production by interacting with JUNB and interferon regulatory factor 4 (IRF4) to regulate gene expression. BATF3-overexpressing T cells lost their exhaustion phenotype upon chronic antigen stimulation; moreover, BATF3-overexpressing CAR-T cells showed increased anti-tumor effects [287]. Similarly, Hogan et al. found that BATF and IRF4 could cooperate to combat T cell exhaustion, and BATF-overexpressing CAR-T cells showed increased survival and expansion [288]. However, it is also necessary to pay attention to the potential risks that may be caused by reprogramming gene expression. For example, BATF3 was previously suggested to drive the development of T cell leukemia by inducing *myc* transcription [289–291]. Recent studies have shown that BATF3 needs to be combined with the biallelic deletion of *Tet2* for tumorigenicity, allowing TET2 to prevent uncontrolled proliferation of BATF3-induced CAR-T cells [292].

Other approaches, such as depleting MDSCs or arming CAR-T cells with pro-inflammatory cytokines (e.g., IL-12 and IL-23), can also prevent exhaustion and improve CAR-T cell function [94, 116, 213]; however, the potential risk of CRS or other systemic toxicities should be considered. Recently, Brudno et al. developed MASTER, a biomaterial that can shorten the tedious and time-consuming process of in vitro CAR-T cell production to a single-day process, allowing the entire activation and CAR transduction process to be completed in patients [293]. The MASTER technique can potentially greatly reduce CAR-T cell preparation time and thus provide treatment opportunities for patients with rapid disease progression. More importantly, CAR-T cells produced using this technique can better maintain their memory phenotype and exhibit superior durability as they would not undergo extensive in vitro expansion. Although all of the aforementioned strategies are promising to address the exhaustion issue in CAR-T cell treatment for solid tumors, most are still in the preclinical stage and require further demonstration in clinical trials.

4. Antigen escape

**Fig. 5:**
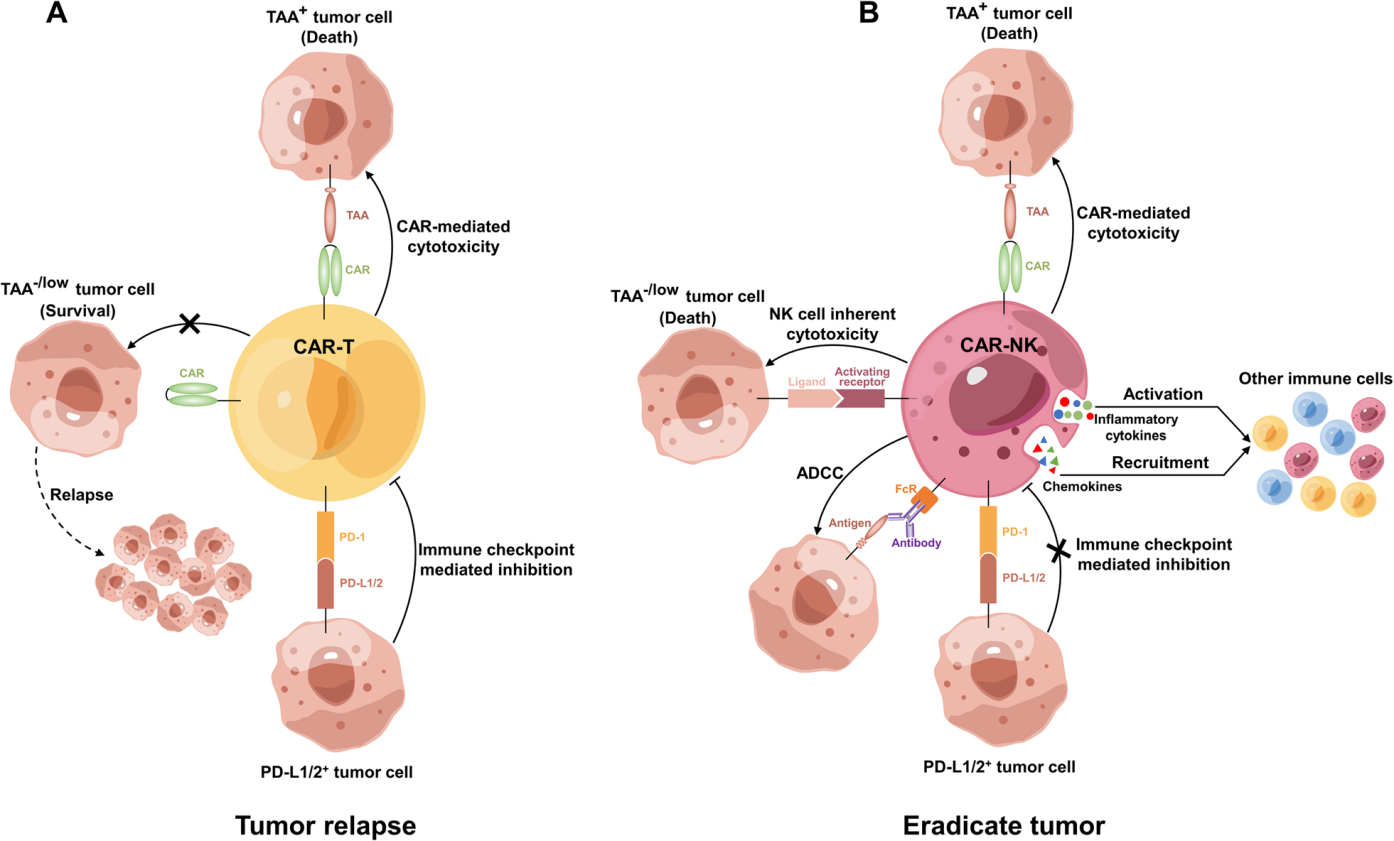
Comparison of CAR-T and CAR-NK cell therapies. **A** CAR-T cells can only kill tumor cells in a CAR-dependent manner, and they cannot kill tumor cells with negative or low TAA expression, leading to tumor escape. Furthermore, CAR-T cells are sensitive to tumor cell-derived PD-L1/2-mediated immunosuppression, which weakens their anti-tumor ability. **B** Except for the CAR-mediated cytotoxicity, CAR-NK cells retain natural recognition and killing functions and can eliminate TAA-negative or low-expressing tumor cells. Furthermore, CAR-NK cells can also kill tumor cells via ADCC and recruit other immune cells, and compared with CAR-T cells, CAR-NK cells are relatively insensitive to PD-L1/2-mediated suppression. TAA, tumor-associated antigen; ADCC, antibody-dependent cellular cytotoxicity

Heterogeneity of antigen density is a typical characteristic of tumors. CAR-T cells are generally ineffective against target cells with low antigen density. Therefore, after receiving CAR-T therapy, a small number of tumor cells with low antigen density often escape and lead to relapse (Fig. 5A). For example, BCMA is the main target of multiple myeloma; however, BCMA-negative relapse (antigen-loss or antigen-low escape) after anti-BCMA CAR-T cell therapy has been observed [294]. This issue can be addressed by employing tandem CAR to simultaneously target multiple targets and eliminate residual tumor cells. Another option is to boost the expression of target antigens, such as by using γ-secretase inhibitors to prevent BCMA degradation in myeloma cells [295].

5. Safety

Apart from the on-target/off-tumor effect, CRS and NT are two defining clinical toxicities associated with CAR-T cell therapy that severely limit its safety [296]. Even the commercially available CAR-T therapies are frequently associated with CRS and NT. In real-world data from Yescarta disclosed by Kite Pharma, 96% of patients experienced and two patients (2%) died of CRS; 76% of patients suffered NT, which led to one fatality [297]. CRS refers to the excessive systemic inflammatory response caused by massive release of cytokines during CAR-T cell therapy, leading to organ damage or even death. Key cytokines related to CRS include IL-6, IFN-γ, TNF, and IL-1 [298, 299]. Elevated IL-6 levels are associated with increased CRS severity [298]. To treat life-threatening CRS during CAR-T cell therapy, the FDA authorized the use of the monoclonal antibody tocilizumab to block IL-6R [300]. Furthermore, other CRS-related cytokine receptor antagonists, such as anakinra, an IL-1 receptor antagonist, are also being investigated to ameliorate the fatal risk associated with CAR-T cell therapy [301]. The pathogenesis of NT is more complicated and less well understood than that of CRS. There are currently no FDA-approved drugs for the prevention or treatment of NT in CAR-T cell therapy. Clinically, corticosteroids can alleviate mild NT; however, continuous exposure for more than 10 days may reduce overall survival in patients with severe NT [302]. Elevated IL-6 levels do not play a critical role in NT, and tocilizumab had no effect on NT despite significantly reducing CRS [303, 304]. Studies have shown that NT severity may be associated with high disease burden before therapy, infusion dose, high expansion of CAR-T cells, high levels or elevated rates of pro-inflammatory cytokines in the blood, endothelial activation, blood–brain barrier injury, and severe CRS [303, 305]. Notably, Bot et al. found that early elevation of IL-15 and granulocyte-macrophage colony-stimulating factor (GM-CSF) was correlated with severe NT, while other pro-inflammatory cytokines were not directly associated [302, 306]. Preclinical research has demonstrated that GM-CSF neutralizing antibodies can prevent CRS and NT in CAR-T cell therapy while also enhancing anti-tumor effects [307, 308]. However, clinical testing is necessary to determine whether GM-CSF antibodies can treat severe NT and ensure the security of CAR-T cell therapy. Other than developing drugs that inhibit CAR-T toxicity, optimizing the design of CAR to improve safety is also a potential approach. The next-generation CAR mentioned above has proposed various strategies that may help solve the issue of safety.

6. GvHD and HvGR

Owing to the high costs and potential risks, CAR-T cell therapy is often used after patients have received unsatisfactory outcomes from radiotherapy, chemotherapy, and other treatments, rather than as first-line treatment. These pretreatments may lower the quantity and quality of T cells in patients, making CAR-T cell preparation impossible [309]. Although allogeneic (also known as universal) CAR-T cells can address the limitation of insufficient T cells, GvHD and HvGR, which are caused by MHC mismatch between the donor and recipient, must be carefully evaluated as they may result in a fatal risk or the elimination of CAR-T cells in vivo. Therefore, the development of allogeneic CAR-T cells requires further genetic modification, such as deletion of TCR and MHC-I, increasing the difficulty of CAR-T cell construction [168, 310]. Furthermore, using human-derived or humanized antibodies for CAR construction [311] or modifying the extracellular hinge and transmembrane domain [312, 313] can reduce the immunogenicity of CAR and help avoid HvGR. Many pharmaceutical companies are actively advancing research on universal CAR-T cells and have reported some promising outcomes. Results of the BCMA-targeting allogeneic CAR-T cell in patients with r/r multiple myeloma were recently reported (NCT04093596) [170]. The CAR-T cells (ALLO-715) used in this study have several advantages, including the use of human-derived scFvs to reduce the immunogenicity and deletion of TCR and CD52 via TALEN technology to avoid GvHD and HvGR. To further improve safety, the extracellular domain of ALLO-715 contains two mimotopes that are susceptible to anti-CD20 monoclonal antibodies, functioning as an off-switch in the presence of rituximab [314, 315]. Among 43 patients with multiple myeloma receiving ALLO-715, only one patient developed grade ≥ 3 CRS, and no patient developed grade ≥ 3 NT. Furthermore, 70.8% of the patients treated with lymphodepletion combined with ALLO-715 showed a response, suggesting good safety and a promising therapeutic effect [170]. Moreover, clinical trials of allogeneic CAR-T cells for other targets and indications have also been conducted, showing good effects in avoiding GvHD and HvGR [171, 174, 316–318]. However, these trials are still in phases 1 and 2, and few of them are suitable for solid tumors; thus, more studies are needed to demonstrate feasibility.

In summary, despite the revolutionary advances made by CAR-T therapy in the treatment of hematologic tumors, many challenges remain to be overcome in the treatment of solid tumors. Many potential solutions have been proposed but have not been validated in clinical trials. Other immune cells that can be modified with CAR should also be taken into consideration in the future considering the unique milieu of solid tumors.

### CAR-NK: powerful innate anti-tumor activity

The drawbacks of CAR-T cell therapy have resulted in an urgent need for new treatments that are convenient, safe, and effective. NK cells, a subset of innate lymphocytes that account for approximately 15% of the total lymphocytes in human peripheral blood, have received considerable attention as a potential alternative platform for CAR engineering therapy in recent years due to their distinctive biological properties. NK cells can identify self- and non-self-signals and killing abnormal cells directly without pre-sensitization. They can also produce a large number of cytokines and chemokines and exert immunomodulatory functions through interactions with DCs and macrophages. These properties make NK cells an ideal tool for cancer immunotherapy.

Similar to CAR-T cell therapy, CAR-NK cell therapy uses CAR molecules that target cancer cells to enable NK cells to more accurately recognize tumor cells. However, CAR-NK has several advantages and has the potential to close the therapy gap left by CAR-T cells in solid tumors (Fig. 1).

#### Advantages

1. Potential for a universal product

NK cells are well suited for the generation of universal cell therapy products due to their homogeneity, low immunogenicity, minimal MHC matching, and lesser risk of GvHD. Therefore, CAR-NK cells can be generated using allogeneic NK cells. This therapy can potentially address the issues with long manufacturing processes and inconsistent quality that limit the application of current CAR-T cell therapies and can potentially reduce treatment costs. Moreover, allogeneic NK cells may be more suitable for cancer immunotherapy because they are not blocked by MHC expressed by tumor cells. Clinical investigations have demonstrated that allogeneic CAR-NK cell therapy is effective and safe [319, 320]. Currently, the sources of NK cells are divided into three categories: (1) Peripheral blood (PB) or umbilical cord blood (UCB). Similar to T cells for CAR-T therapy, NK cells can also be isolated from PB. However, PB is not the optimal cell source because it contains few NK cells. In contrast to PB, UCB contains a large amount of NK cells with greater proliferation potential than those from PB, making it a more feasible source for creating CAR-NK cells [321]. Clinical trials for CAR-NK cells generated from UCB are currently being conducted and showed good safety and efficacy [319, 322]. (2) Stem cells. NK cells can be generated by inducing differentiation of human embryonic stem cells (hESCs) or induced pluripotent stem cells (iPSCs) [323, 324], and they have the same therapeutic potential as adult NK cells. (3) Cell lines. NK cell lines (such as NK92 and YT) can be used to construct CAR-NK cells. After in vitro irradiation, such CAR-NK cell lines lose tumorigenicity but retain the capacity to specifically target and destroy tumor cells [325], which can substantially lower treatment costs when used broadly.

2. Safety

NK cells do not secrete IL-1 and IL-6, which may cause CRS, because their cytokine profiles differ from those of T cells, making CAR-NK therapy less likely to cause fatal side effects than CAR-T therapy. This safety benefit has been validated by the results of numerous clinical trials showing that CAR-NK cell therapies are not associated with CRS or NT [319, 320]. Furthermore, owing to their short lifespan and lack of proliferative ability, CAR-NK cells do not remain in the body for a long time. This may reduce the effectiveness of CAR-NK therapy, but from the perspective of safety, this feature avoids the potential hazards associated with the long-term existence of genetically modified immune cells in the body.

3. Cytotoxicity

Compared with CAR-T cells, CAR-NK cells exhibit a greater variety of strategies to attack tumor cells (Fig. 5B). First, NK cells not only directly lyse tumor cells by releasing perforin and granzymes but also recruit other immune cells to participate in anti-tumor activities by secreting cytokines and chemokines. Second, CAR-NK cells may be more effective than CAR-T cells in destroying PD-1-negative malignancies. The immune checkpoint PD-1 is consistently upregulated in activated T cells, which in turn limits their activity. However, a recent study demonstrated that PD-1 is not naturally expressed by NK cells; rather, they acquire it through trogocytosis from tumor cells, resulting in a functional inhibition. Consequently, NK cells can exert a stronger anti-tumor effect than T cells upon encountering PD-1-negative tumors because the inhibitory circuit mediated by trogocytosed PD-1 is absent [326]. Third, NK cells express Fc receptors (CD16) and are among the strongest cells in mediating ADCC functions, suggesting great potential in combination with antibody drugs to fight tumors. In 2020, Fate Therapeutics Inc. announced that its anti-CD19 CAR-NK cells showed stronger cytotoxicity against CD20^+^ lymphoma cells in patients with r/r B cell lymphoma (BCL) when combined with the CD20 antibody–drug rituximab. This suggests that the combination of antibody drugs and CAR-NK therapy may have a synergistic effect [320, 327, 328]. Finally, in addition to killing target cells in a CAR-dependent manner, CAR-NK cells also retain their inherent cytotoxicity, which is partly dependent on activating receptors such as NKG2D. As stressed cancer cells always express more of the activating ligands, CAR-NK cells may have a superior ability to kill tumor cells with low antigen density and high activating ligand expression and can thus better prevent treatment failure caused by antigen heterogeneity [329]. Clinical trials have demonstrated that among four patients with prior CAR-T therapy, two (50%) who received CD19 CAR-NK cell therapy achieved CR [328], indicating that CAR-NK cell therapy may be effective even when CAR-T cell therapy fails. However, it should be noted that this target-independent cytotoxicity may also introduce potential toxicity risks, which may be amplified when large infusions are required in clinical trials.

#### Research progress

Currently, CAR-NK therapy is in the early stages of research. Although the number of relevant clinical trials has increased rapidly in recent years, almost no clinical data have been published. CAR-NK clinical research mainly focuses on hematological tumors while also attempting to treat solid tumors. The majority of these clinical studies are only in phase 1, with a few in phase 2 (Fig. 2A). Hematoma-associated antigens (such as CD19, CD33, BCMA, and CD22) and solid tumor-related antigens (such as NKG2D ligand, PD-L1, ROBO1, and 5T4) are commonly employed in such clinical trials (Fig. 2C).

Published data indicate that CAR-NK cell therapy has clinical application potential. According to the data of a phase 1/2 clinical trial published by Rezvani et al. in 2020 [319], 8 (73%) out of 11 patients with CD19-positive lymphoid tumors responded to allogeneic UCB-derived CD19 CAR-NK cell therapy, and seven of them achieved complete remission. Notably, none of the patients receiving this treatment suffered CRS, NT, or GvHD. The allogeneic iPSC-derived CAR-NK cell therapy FT596 developed by Fate Therapeutics Inc. similarly demonstrated potent tumor eradication in patients with r/r BCL without side effects of any grade [320]. Furthermore, results from a recently published phase 1 clinical study regarding local application of HER2-specific CAR-NK92 cells for the treatment of recurrent glioblastoma, the primary objective of which was to evaluate the safety and tolerability 24 weeks post-injection, revealed that a dose of 1 × 10^8^ irradiated CAR-NK92 cells did not cause any side effects [330]. These results show the effectiveness and safety of CAR-NK cell therapy, as well as the feasibility of generating CAR-NK cells from allogeneic NK cells. However, the clinical efficacy of CAR-NK cell therapy needs to be further evaluated.

#### Challenges and potential solutions

Although CAR-NK cell therapy holds considerable potential, there are several important challenges that need to be addressed before it can be widely used in clinical settings (Fig. 1).

1. Poor in vivo persistence

CAR-NK cells cannot persist in the body for a long period of time due to their short lifespan (approximately 2 weeks) and poor capacity for expansion in the recipient [331]. This is a major obstacle that CAR-NK cell therapy must overcome, as poor persistence is always associated with an unfavorable prognosis. Although CAR-NK cells made from cell lines can proliferate indefinitely, they must be irradiated prior to infusion due to their tumorigenicity, which makes CAR-NK incapable of proliferation and gives them poor persistence in vivo. In a clinical trial of CD33-targeting CAR-NK cell therapy employing NK92 cell lines, patients with acute myeloid leukemia (AML) received up to 5 × 10^9^ CAR-NK92 cells without experiencing any substantial adverse effects or grade 3–4 toxicity in 2018. However, these CAR-NK92 cells did not offer a long-lasting remission, as they became undetectable a week after infusion [332].

Strategies to boost NK cell activation, such as knocking out or blocking inhibitory receptors on NK cells or increasing the expression of activated ligands on target cells, can compensate for poor persistence. Brien et al. demonstrated that combining a HDAC inhibitor and a PD-1/PD-L1 blocker improved the anti-tumor effect of anti-GD2 CAR-NK in a mouse neuroblastoma model [333]. TGF-β markedly inhibits the NK cell function. By introducing a chimera comprising the extracellular and transmembrane domains of the type II TGF-β receptor (TGFBR2) fused to the NKG2D intracellular domain, NK92 cells can be induced to more effectively infiltrate and attack HCC xenografts [334]. Moreover, many cytokines (such as IL-2, IL-15, and IFN-γ) can be used to promote NK cell proliferation. Bayle et al. introduced iMC into CAR-NK cells to promote NK cell function and further enhance CAR-NK cell proliferation by coupling them with ectopic IL-15 [335]. Similarly, CAR-NK cells expressing IL-15 have demonstrated long-term persistence and potent anti-tumor activity in clinical trials [319, 336]. Therefore, combining cytokines with CAR-NK cells may improve their persistence; however, attention should be paid to potential side effects and exhaustion caused by long-term exposure to cytokines [337].

2. Insufficient infiltration

Similar to CAR-T cells, CAR-NK cells face difficulty infiltrating solid tumors, and many attempts have been made to improve the infiltration of CAR-NK cells. Temme et al. discovered that anti-EGFRvIII CAR-NK cells overexpressing CXCR4 showed increased chemotaxis to U87-MG cells producing CXCL12 (a ligand for CXCR4) [338]. Similarly, anti-NKG2D CAR-NK cells overexpressing CXCR1 also showed stronger chemotaxis and infiltration to hypopharyngeal and ovarian cancer cells in a mouse model [339]. Moreover, the addition of OVs may enhance CAR-NK cell infiltration, although more research is needed [340]. Furthermore, local injection strategies are also used to circumvent the major barriers limiting infiltration and may improve the efficacy of CAR-NK cell therapy. In an in situ glioblastoma xenograft mouse model, Yu et al. intracranially injected EGFR-targeting CAR-NK92 cells into mouse brains and found that they effectively inhibited tumor development and significantly improved the survival rate of tumor-bearing mice [341]. Similarly, the local injection of HER2-targeting CAR-NK92 cells into glioblastoma mouse models resulted in complete tumor regression in the majority of mice [342].

3. Limited supply of NK cells

In contrast to T cells, the number of NK cells in PB is very low and their proliferation ability is poor; therefore, other sources of NK cells must be used to meet the demand for CAR-NK cells preparation. NK cells from allogeneic peripheral blood mononuclear cells (PBMC) can be considered because they are MHC-independent, which expands the pool of NK cell sources [343]. However, to prevent GvHD, NK cells must be completely separated from T cells.

NK cells are abundant in UCB and have a higher proliferative capacity than those in PB [343, 344]. UCB-derived CAR-NK cell therapy has entered clinical trials and demonstrated promising results. In a phase 1/2 clinical trial, 7 out of 11 patients with CD19-positive tumors who received UCB-derived anti-CD19 CAR-NK achieved complete remission, and the infused CAR-NK cells were retained for at least 12 months in vivo without CRS, NT, or GvHD (NCT03056339) [319]. However, primary NK cells derived from both UCB and PBMC exhibit heterogeneity between different donors, making it challenging to achieve uniform CAR-NK cell quality. In contrast, hESCs and iPSCs can be used to generate large numbers of NK cells with uniform quality [343]; however, the procedure is time-consuming and potential risks exist due to the tumorigenicity of iPSCs.

NK cell lines are an attractive source of NK cells due to their capacity for indefinitely proliferation and low culturing costs, which enable the generation of numerous homogeneous CAR-NK products [332]. However, owing to safety issues associated with their malignant origin, CAR-NK cells produced from cell lines must be irradiated before being administered to patients, which can have a severe impact on their long-term in vivo persistence and overall therapeutic potential. This issue has not yet been addressed. Another drawback is that CD56^bright^ NK cell lines (such as NK92) lack the potential to mediate cell death via ADCC as they lack CD16 [345].

4. Lack of CAR optimization for NK cells

The current CAR structure in CAR-NK cell therapy almost completely misappropriates the design of CAR-T cells, which may not be the best choice. For example, the most commonly used costimulatory domains in CAR-T cells are CD28 and 4-1BB, of which CD28 is the activating receptor of NK cells [346], but the role of 4-1BB in NK cells is controversial. Some studies have suggested that 4-1BB can stimulate NK cell proliferation and cytokine secretion [347], while others have shown that it acts as an inhibitory receptor in human NK cells [348, 349]. In CAR-T cell therapy, although third-generation CAR did not outperform second-generation CAR, Guo et al. reported that the third-generation CAR-NK92 cells exhibited stronger cytotoxicity and proliferation than the first- and second-generation CAR-NK92 cells against αFR^+^ ovarian cancer cells [350]. Furthermore, studies have also shown that replacing CD3ζ with the NK activating receptor signaling adaptor molecule DAP12 can endow CAR-NK cells with a stronger anti-tumor activity [351]. Therefore, CAR should be tailored according to the characteristics of NK cells rather than simply by copying CAR-T cells. Only with this approach can CAR-NK therapy exert the greatest anti-tumor effect.

5. Challenging gene transduction

Achieving gene transduction is substantially more challenging for NK cells than it is for T cells. The typical lentiviral vector used in CAR-T cell therapy has a transfection efficiency for NK cells that frequently falls below 20% [352]. One study showed that the treatment of NK cells with rosuvastatin to upregulate low-density lipoprotein receptor enhanced the transduction efficiency of vesicular stomatitis virus glycoprotein (VSV-G) pseudotyped lentivirus-based vectors without affecting cytotoxicity [353], but this approach has not been attempted with a CAR-NK cell preparation. Although the retrovirus vector has a higher transduction efficiency than the lentivirus, there is a risk of insertion mutation [354]. mRNA electroporation can achieve high transduction efficiency. Xu et al. constructed anti-NKG2D CAR-NK cells on PBMC-derived NK cells via mRNA electroporation and demonstrated their anti-tumor effects in vitro, in tumor-bearing mice, and in patients with colorectal cancer [355]. However, because mRNA electroporation can only mediate the non-integrated, transient expression, CAR-NK cells generated by this method must be infused multiple times, which increases the workload and the demand for NK cells. Other gene transduction methods that have been used successfully in CAR-T cell therapy, such as transposon system and adeno-associated viral (AAV) transduction, still lack empirical support in CAR-NK cell production. Therefore, the optimization of gene transduction for NK cells determines whether high CAR-positive CAR-NK cells can be obtained and requires more attention and investigation.

6. Sensitivity to freezing and thawing

Primary NK cells are susceptible to the freeze–thaw process, and their survival rate and cytotoxicity after resuscitation are greatly reduced [356]. Consequently, many NK cells can be lost, exacerbating the problem of insufficient NK cells. The addition of IL-2 can partially restore the vitality of NK cells, but improvement of freezing tolerance is still necessary.

Overall, CAR-NK cell therapy, which is considered to be the second-most anticipated ACT after CAR-T cell therapy, has advanced significantly due to recently reported clinical and preclinical data. However, it is still limited to preclinical studies, and clinical studies are not extensive. More clinical evidence is urgently necessary to establish both its ultimate efficacy and any potential adverse effects in the human body.

### CAR-macrophage: future nemesis of solid tumors

The application of CAR to macrophages, which began after T and NK cell applications, has gradually attracted attention in recent years [357]. Macrophages, which play critical roles in phagocytosis, cytokine secretion, and antigen presentation, are an important component of the innate immune system and serve as the key hub connecting innate and adaptive immunity [358]. Macrophages can be divided into the functionally opposing M1 and M2 subgroups. M1 macrophages are pro-inflammatory and have anti-tumor properties [359, 360], whereas M2 macrophages suppress immune responses and promote angiogenesis. Macrophages are highly plastic and can thus exhibit the M1 or M2 phenotype in response to any pathological conditions [361, 362]. Tumor-associated macrophages (TAMs), characterized by an M2-polarized phenotype, are critical for tumor growth and metastasis and have been recognized as attractive therapeutic targets for cancer [363, 364]. TAMs constitute approximately 50% of tumor mass, indicating that macrophages have the highest infiltration rate among all immune cells and can easily enter the interior of solid tumors where T cells and NK cells cannot infiltrate [365]. Furthermore, the reinfusion of modified human bone marrow-derived macrophages has been shown to induce A549 xenograft regression with good in vivo safety [366], suggesting that ACT based on macrophages also has a broad range of applications, particularly for the treatment of solid tumors. Considering this, researchers are attempting to engineer macrophages with CAR to fight tumors.

**Fig. 6:**
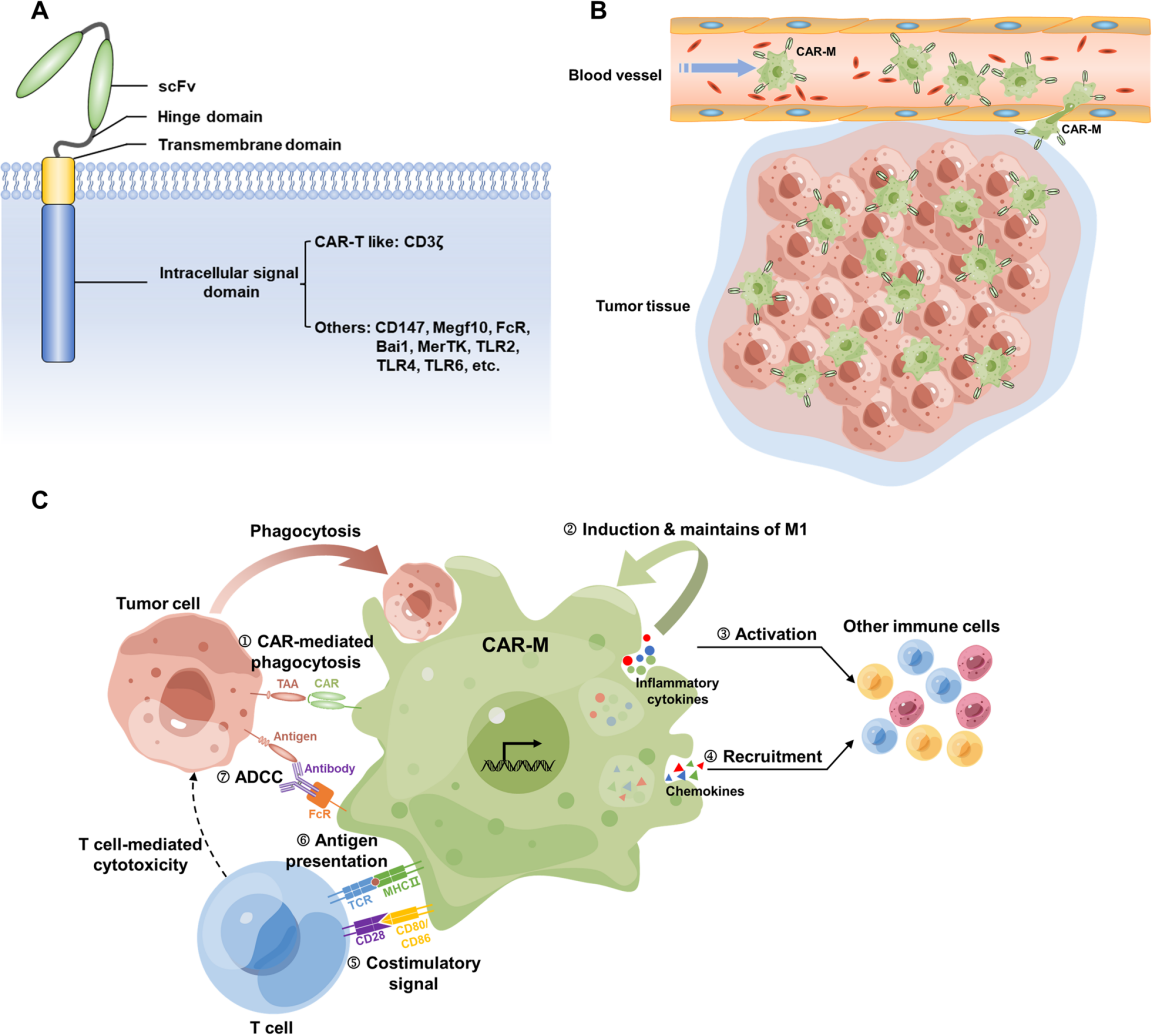
CAR architecture and the anti-tumor modes of CAR-M cells. **A** The architecture of CAR in CAR-M cells is similar to that in CAR-T cells, but the intracellular signaling domain is more abundant, mainly including CAR-T like and other intracellular signaling domains. **B** CAR-M cells are easily delivered to various tissues through blood vessels and can infiltrate solid tumors. **C** CAR-M cells can mediate tumor cell phagocytosis in a CAR-dependent and CAR-independent manner. Furthermore, CAR-M cell possess antigen presentation, costimulatory signaling, and cytokine secretion abilities to recruit other immune cells to participate in anti-tumor response. FcR, Fc receptor; TLR, toll-like receptor; and TCR, T cell receptor

Similar to CAR-T and CAR-NK, CAR-M therapy refers to the modification of macrophages with specific CARs to improve the phagocytic activity and antigen presentation of macrophages toward tumors [367]. The CAR structure is composed of an extracellular recognition domain, a transmembrane region, and an intracellular signaling domain (Fig. 6A). Intracellular signaling domains commonly used in CAR-M research include CD3, CD147, Megf10, and FcR [368–371]. In 2018, Vale et al. developed the first CAR-M cells, which were originally known as CAR-phagocytes (CAR-Ps), by using a lentiviral vector to introduce a novel CAR with Megf10 or FcRγ as the intracellular domain into the mouse macrophage cell line J774A.1. Moreover, they discovered that these CAR-Ps exhibited specific engulfment of whole human cancer cells, particularly when a tandem PI3K p85 subunit was also included in the CAR [368]. This study, despite focusing only on how CAR affects phagocytosis and omitting other crucial anti-tumor effects handled by macrophages, opens a new chapter in the study of CAR-based immunotherapy by suggesting, for the first time, that CAR expression in phagocytic cells is sufficient to stimulate the targeted engulfment and elimination of cancer cells.

#### Advantages

1. TME improvement

Relying on a single type of immune cell to achieve a long-lasting and effective anti-tumor response is unfeasible; instead, a variety of immune cells operating in concert can have a better effect. Macrophages have a strong potential to modify the TME because they can interact with nearly all local immune cells (such as T cells, NK cells, and DCs) (Fig. 6C). In addition to enhancing the phagocytosis of target cells, the antigen-presenting ability of CAR-M cells enables them to transmit information regarding target cells to other immune cells, thereby mobilizing various immune cells to participate in tumor-killing activities. Furthermore, CAR-M cells can induce M2 macrophages to polarize toward M1, greatly reduce the proportion of TAMs in the TME, and secrete pro-inflammatory cytokines to modify the immunosuppressive TME, all of which have a positive effect on cancer therapy [369]. In 2020, Gill et al. used an incompletely replicated chimeric adenovirus vector (Ad5F35) to introduce a HER2-targeting CAR into macrophages. They subsequently discovered that HER2-targeting CAR-M cells not only exhibited a specific killing ability in vitro and in vivo, but also induced a pro-inflammatory TME and boosted anti-tumor T cell activity by producing pro-inflammatory cytokines and chemokines, converting bystander M2 macrophages into M1, and upregulating antigen-presenting machinery [369]. This study demonstrates the potential of CAR-M cells to improve the TME.

2. Extremely strong infiltration

T and NK cells only exhibit limited tumor infiltration due to the dense ECM surrounding tumor cells, whereas macrophages are naturally recruited to the TME and thus are the most prevalent immune cells in tumor tissues [365] (Fig. 6B). This is because macrophages can secrete matrix metalloproteinases (MMPs), key enzymes that degrade the ECM. Shen et al. constructed CAR-M cells using CD147 as the intracellular signaling domain (CAR-147 M) and discovered that when co-cultured with target cells, these CAR-147 M cells exhibited significantly increased MMP expression. Despite not having an impact on tumor cell proliferation in vitro, CAR-147 M cells rapidly accumulated in the tumor site after being infused in vivo, reduced tumor collagen deposition, and encouraged T cell infiltration, which resulted in significant tumor suppression. This implies that CAR-M cells secrete proteases that not only enable them to infiltrate effectively but also encourage the infiltration of other immune cells [370].

3 Safety

CAR-M cells may be safer than other CAR-based cell therapies. Similar to CAR-NK cells, CAR-M cells have a limited circulation period in the body, which can avoid the potential harm that genetically engineered cells may bring. Furthermore, there is no GvHD risk or requirement for MHC matching when using allogeneic macrophages. Moreover, CAR-M cells may not cause serious or fatal CRS. Shen et al. reported that levels of CRS-related pro-inflammatory cytokines (such as IL-6, IL-1β, and TNF-α) were significantly reduced in the PB of CAR-147 M-treated mice [370].

4. High universality for therapy

As allogeneic administration of macrophages does not result in GvHD, allogeneic macrophages can be used to create universal CAR-M cells. The use of CAR-expressing macrophages produced from iPSCs (CAR-iMs) in tumor treatment was first reported by Zhang et al. in 2020, who discovered that CAR-iMs are more polarized toward the M2 phenotype in the absence of antigens. However, following antigen stimulation, CAR-iMs exhibited enhanced phagocytosis and M1 phenotype preference. In various mouse hematological and solid tumor models, CAR-iMs demonstrated effective anticancer activity without exhibiting notable side effects [371]. This study demonstrated the potential of CAR-M therapy as a universal treatment and raised the possibility that iPSCs could be used as a cell source for CAR-M therapy in the future.

5. Multiple killing modes

In addition to antigen-specific phagocytosis, CAR-M cells have the capacity for antigen-independent phagocytosis. Furthermore, the antigen-presenting ability of CAR-M cells can facilitate the killing of tumor cells mediated by other immune cells, facilitating more comprehensive killing of highly heterogeneous tumor cells [372] (Fig. 6C). Gill et al. reported that chimeric adenovirus vectors can not only successfully introduce CAR into macrophages but also confer the M1 phenotype of CAR-M cells and prevent M2 induction [369], potentially enhancing the non-specific killing ability of CAR-M cells. Notably, they found that CAR-M cells can present antigenic epitopes to T cells, enabling tumor-specific T cells to activate and proliferate. These diverse tumor-killing modes aid in the complete eradication of tumor cells as well as the prevention of relapse.

#### Research progress

Currently, only two CAR-M related studies are registered on *Clinicaltrials.gov* (Table 2). The first is CT-0508 (HER2-targeting CAR-M cells derived from chimeric adenoviral vector Ad5f35-transduced iPSCs, NCT04660929) from Carisma Therapeutics, which is scheduled to complete clinical trials in 2023. CT-0508 is the first CAR-M therapy approved for clinical trials, marking the beginning of a new era of cancer therapy. Furthermore, the FDA granted CT-0508 Fast Track designation in September 2021, demonstrating the urgent need for CAR-M therapy development. The other study is an in vitro trial in which the anti-tumor activity of CAR-M cells is being examined in tumor samples collected from patients (NCT05007379). As shown by the scarcity of studies, CAR-M therapy is still in its infancy. More clinical data are urgently needed to confirm the feasibility of CAR-M therapy, and the results of the CT-0508 clinical trial are greatly anticipated.

**Table 2:** Summary of CAR-M, CAR-γδT, and CAR-NKT in clinical trials of cancer therapy

NCT number	Status	Conditions	Target	Phase	Number enrolled	Study start	Study completion
CAR-M							
NCT04660929	Recruiting	HER2 overexpressing solid tumors	HER2	Phase 1	48	2021, Feb	2024, Dec
NCT05007379	Not yet recruiting	Breast cancer	HER2	-	100	2021, Sep	2023, Sep
CAR-γδT							
NCT02656147	Unknown	Leukemia, lymphoma	CD19	Phase 1	48	2017, Oct	2020, Apr
NCT04796441	Unknown	AML	CD19	Not Applicable	20	2020, Dec	2022, Feb
NCT05554939	Recruiting	Non-Hodgkin’s lymphoma	CD19	Phase 1/2	30	2022.Dec	2025, Dec
NCT04735471	Recruiting	B cell malignancies	CD20	Phase 1	78	2021, Mar	2024, Mar
NCT04911478	Enrolling by invitation	Lymphoma	CD20	-	50	2022, Feb	2039, Aug
NCT04702841	Unknown	Relapsed and refractory CD7 positive T cell-derived malignant tumors	CD7	Early Phase 1	8	2020, Jun	2022, Dec
NCT05388305	Recruiting	AML	CD123	Not Applicable	30	2022, Apr	2023, May
NCT04107142	Unknown	Relapsed or refractory solid tumor	NKG2DL	Phase 1	10	2019, Dec	2021, Mar
NCT05302037	Not yet recruiting	Advanced cancers	NKG2DL	Phase 1	9	2022, Apr	2023, Dec
CAR-NKT							
NCT03774654	Recruiting	Relapsed or refractory B cell malignancies	CD19	Phase 1	48	2020, Jun	2035, Mar
NCT04814004	Recruiting	Relapsed/Refractory/High-risk B cell Tumors	CD19	Phase 1	20	2021, Mar	2024, Apr
NCT05487651	Recruiting	B cell malignancies	CD19	Phase 1	36	2022, Oct	2024, Dec
NCT02439788	Withdrawn	Neuroblastoma	GD2	Phase 1	0	2017, Aug	2030, Oct
NCT03294954	Active, not recruiting	Neuroblastoma	GD2	Phase 1	36	2018, Jan	2034, Aug

Data were obtained from clinicaltrials.gov and updated until July 2023

#### Challenges and potential solutions

Clinical trials of CAR-M therapy are in their early stages, and numerous potential issues remain to be handled and verified.

1. Potential “mutiny”

Although CAR-M cells have demonstrated promise in preclinical investigations, their performance in the complex interior milieu of the human body could be complicated by their highly flexible nature. Limited clinical data are available to demonstrate the safety and effectiveness of CAR-M therapy in vivo. One potential risk is that CAR-M cells could lose their anti-tumor properties and potentially transform into TAMs that accelerate tumor growth, particularly in the suppressive TME. Multiple remedies have been proposed to address this issue, including targeting the crucial pathways that induce and/or regulate macrophage polarization, such as a CSF1R blockade [373, 374] or co-expression of IFN-γ [375], which may be beneficial for preserving the phenotypic and anticancer efficacy of CAR-M cells.

2. Limited availability

CAR-M therapy requires a large number of cells and frequent infusions. Consequently, macrophage availability presents a challenge because human primary macrophages make up a relatively small proportion of PB and cannot proliferate either in vitro or in vivo. However, allogeneic macrophages can be employed because they are less likely to result in GvHD. iPSCs are a good alternative and have been tested in a clinical trial [371], though concern exists over their potential tumorigenicity.

3. Challenges in gene transduction

Macrophages act as first responders to defend against viral infection; therefore, they are resistant to the typical viral vectors commonly used in gene and cell therapies, which makes creating CAR-M cells more difficult. Moreover, because macrophages are highly plastic innate immune cells, the gene transduction method used to construct CAR-M cells must be carefully selected to avoid affecting their phenotype. Currently, the chimeric adenovirus vector Ad5F35 constructed by Gill et al. has demonstrated good potential for gene transduction in macrophages. It not only efficiently introduces foreign genes but also encourages CAR-M cells to polarize toward the M1 phenotype [369]. Notably, nanobiotechnology offers a method to construct CAR-M cells in vivo. Kang et al. successfully delivered the genes encoding CAR and IFN-γ to macrophages in tumor tissues via intratumoral injection of macrophage-targeting polymer nanocarriers and discovered that CAR-M cells produced using this method had anti-tumor abilities as well as the capacity to alter the TME and boost overall anti-tumor immune response [376]. This method, if it can meet the requirements for clinical application, will assist in avoiding the difficult and expensive ex vivo CAR-M cell manufacturing processes. Moreover, CAR-M cells produced in vivo using this method bypass infiltration, potentially leading to a better anti-tumor effect.

4. Potential toxicity of in vivo CAR-M cell dispersion

The migration characteristics of the imported macrophages in vivo will seriously affect their therapeutic effect. According to reports, the infused CAR-M cells travel through the lungs and then stay mainly in the liver, where they are even more enriched than in tumors [377]. This diffusion property of CAR-M cells not only negatively impacts treatment outcomes but also raises the possibility of liver injury. However, these limitations of CAR-M therapy may be circumvented via co-expression of a particular chemokine receptor to drive CAR-M cell migration toward the tumor or by local injection of CAR-M cells to avoid the in vivo circulation process.

5. Inadequate CAR optimization for macrophages

Macrophages differ significantly from NK and T cells in function and activation pattern, necessitating optimization of the CAR structure to better match macrophages. Although CD3ζ is frequently used and has been shown to boost specific phagocytosis, pro-inflammatory cytokine production, and antigen presentation of CAR-M cells, the efficacy and persistence of CD3ζ-based CAR-M cells still require improvement. Through research using RAW264.7 cells, Zhu et al. discovered that employing Mer receptor tyrosine kinase (MerTK) as the intracellular domain confers macrophages with potent phagocytic abilities toward tumor cells, whereas using TLR2, TLR4, TLR6, and CD3ζ had relatively little effect [378]. However, Vale et al. reported that CAR using Megf10 or FcRγ, but not Bai1 or MerTK, as intracellular domains endowed J774A.1 macrophages with phagocytic capabilities similar to those achieved using CD3ζ. Furthermore, the addition of a random PI3K-recruiting motif significantly enhanced the antigen-specific phagocytosis activity of such CAR-M cells. Although some of these data are debatable and based on murine cells, they do indicate that it may be possible to alter the CAR structure to increase CAR-M cell efficacy. Future research should focus on screening the proper intracellular domains and optimizing CAR structure to more precisely assemble human primary macrophages [368].

Overall, CAR-M cells have shown effective anti-tumor effects in preclinical studies and are superior to CAR-T and CAR-NK cells in some aspects, particularly in their capacity to infiltrate tumors. Although further clinical research into effectiveness and safety is still necessary, the development of CAR-M therapy has nonetheless created new opportunities for the treatment of solid tumors.

### CAR-γδT: a promising candidate for allogeneic cell therapy

Although αβT cells are the T cell subset primarily used in CAR-T cell therapy, γδT cells share various unique features that could confer new capabilities to CAR-T cells. γδT cells, a critical component of the innate immune system, account for approximately 1–5% of PB T cells and are mainly divided into three subtypes: Vδ1, Vδ2, and Vδ3. With both innate and adaptive characteristics in immune response, γδT cells can rapidly recognize and respond to non-MHC-restricted tumor antigens. A significant association between tumor-infiltrating γδT cells and favorable prognosis was discovered by Gentles et al. when they analyzed the gene expression signature of approximately 18,000 samples from 39 types of malignant tumors [379]. Therefore, anti-tumor treatments based on unmodified γδT cells have long received attention in clinical research. For example, in a clinical trial examining adoptive transfer of allogeneic γδT cells reported by Yin et al. in 2021, the infusion of γδT cells significantly prolonged the survival of patients with liver and lung cancers [380]. However, compared to the success of CAR-T in hematological tumors, the effectiveness of γδT adoptive therapy remains unsatisfactory. Therefore, several novel approaches have been developed to address the shortcomings of γδT cell therapy, the most promising of which involves engineering γδT cells with CAR. The first CAR-γδT cell therapy was created in 2004 by Rossig et al., who produced CAR-γδT cells by stimulating PBMC-derived γδT cells with aminobisphosphonates in vitro and delivering first-generation GD2- or CD19-specific CARs into γδT cells using retrovirus vectors. When co-cultured with the corresponding target cells, these CAR-γδT cells showed specific cytolysis along with elevated levels of IFN-γ and the T cell activation marker CD69 [381]. This study demonstrated the anti-tumor potential of CAR-γδT cells for the first time.

#### Advantages

1. Multiple killing modes

**Fig. 7:**
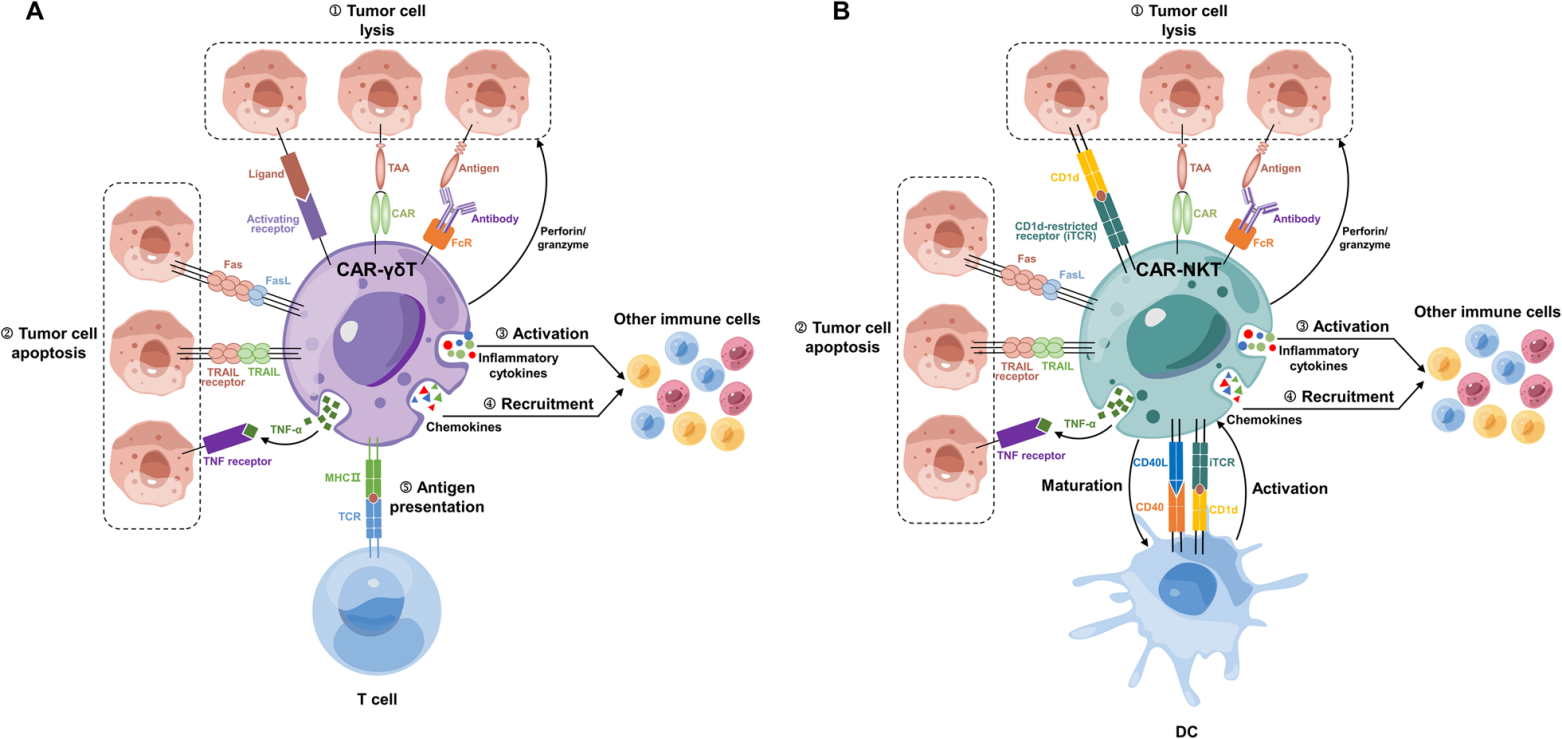
Anti-tumor mode of CAR-γδT and CAR-NKT cell therapies. Both CAR-γδT and CAR-NKT cells can kill tumor cells through CAR-, ADCC-, Fas/FasL-, TRAIL-, and TNFR-mediated ways and can mobilize other immune cells by secreting cytokines. CAR-γδT cells can also be activated with select receptors, whereas the activating receptor of CAR-NKT cells is limited to CD1d. Furthermore, CAR-γδT cells can present antigens to T cells, and CAR-NKT cells can promote DC maturation and be activated by DCs. TAA, tumor-associated antigen; ADCC, FasL, Fas ligand; TRAILR, TRAIL receptor; TNFR, TNF receptor; TCR, T cell receptor; iTCR, invariant T cell receptor; CD40L, CD40 ligand; and DC, dendritic cell

In addition to CAR-mediated cytotoxicity, γδT cells can also recognize tumor cells through intrinsic receptors (Vδ1TCR, CD16, NKG2D, and NKp30) and activate various natural tumor-killing pathways, such as perforin/granzyme-dependent cytotoxicity, CD16-mediated ADCC, and TRAIL/FASL-triggered apoptosis [382] (Fig. 7A). Additionally, γδT cells can also serve as APCs that present tumor antigens to αβT cells. Anderson et al. found that anti-GD2 CAR-γδT cells produced from PB not only specifically destroyed the GD2^+^ LAN1 cells but also retained the ability to endocytose and present tumor antigens to αβT cells, leading to the clonal expansion of αβT cells [383].

2. Universal applicability

As MHC non-restricted lymphocytes, γδT cells differ from αβT cells in that they do not cause GvHD, making them ideal for the development of universal CAR-γδT therapy [384]. In a clinical experiment using allogeneic γδT cells to treat liver and lung cancer, Yin et al. discovered that γδT cells have good safety in vivo [380].

3. Effective infiltration and resistance to hypoxia

γδT cells, particularly the Vδ1 subtype, have a homing advantage over αβT cells and are thus better able to infiltrate tumors, especially those with a hypoxic TME [385, 386]. Additionally, γδT cells may function more effectively in the hypoxic TME because their cytotoxicity, as well as their secretion of MIP1, RANTES, and CD40L, can be enhanced by hypoxia. However, tumor cells also employ strategies such as secreting soluble MHC-I-related molecules to avoid γδT cell-mediated killing [387], which must be overcome by developing more potent γδT immunotherapies [388]. Anderson et al. reported that engineering γδT cells with CAR can increase their cytotoxicity while retaining their ability to migrate toward tumor cells and cross-present antigens [383]. These studies implying that incorporating CAR into γδT cells is feasible and may be effective in treating hypoxic solid tumors.

4. TME amelioration

Activated γδT cells can secrete various pro-inflammatory cytokines and chemokines to create an inflammatory environment (Fig. 7A). Additionally, γδT cells serve as a bridge between innate immunity and adaptive immunity, promoting the maturation of DCs, activating NK cells, promoting antibody production in B cells, and improving the humoral immune response. Moreover, γδT cells can serve as APCs and mobilize bystander immune cells to attack tumor cells [386] (Fig. 7A). For example, Anderson et al. found that anti-GD2 CAR-γδT cells functioned as APCs, processing and presenting the antigen to αβT cells while simultaneously inducing their clonal expansion [383].

#### Research progress

Although the preclinical results of CAR-γδT are impressive, there are few clinical trials, and most are focused on hematological tumors (Fig. 2A, Table 2). The most advanced current option is ADI-001, an allogeneic CD20-targeting CAR-γδT cell therapy that was developed to treat B cell non-Hodgkin’s lymphoma [389]. ADI-001 is currently being investigated in a phase 1 clinical trial to evaluate its safety and efficacy in monotherapy or in combination with IL-2 [390]. In June 2022, Adicet Bio released partial clinical data for ADI-001 (NCT04735471), demonstrating a 78% ORR and CR rate in nine patients who had received multiple prior treatments, with no patient experiencing grade ≥ 3 CRS, ICANS, or GvHD. Notably, the ORR and CR rate for the four patients who had received prior anti-CD19 CAR-T therapy was both 100% [391]. Considering its promising efficacy and safety, the FDA has granted Fast Track designation to ADI-001. Furthermore, clinical trials of CAR-γδT cells targeting CD123 (NCT05388305), CD19 (NCT04796441, NCT02656147, NCT05554939), CD7 (NCT04702841), and NKG2D ligands (NCT04107142, NCT05302037) are also in progress; however, clinical data have not yet been published.

#### Challenges and potential solutions

1. Limited γδT cell quantity

In clinical trials of CAR-T therapy, the usual dose is 1–5 × 10^6^ CAR-positive αβT cells/kg, with approximately 10^8^ cells per infusion per adult [392]. However, it is difficult to achieve this dose of CAR-γδT cells due to the scarcity of γδT cells in PB and the challenge of in vitro expansion. Conventional methods for expanding αβT cells, such as anti-CD3/CD28 antibodies and IL-2, usually do not result in the effective expansion of γδT cells, which is a problem that must be solved for the clinical application of CAR-γδT cell therapy. Therefore, many preclinical studies have focused on addressing the problem of γδT cell expansion.

Aminobisphosphonates are frequently used to induce γδT cell expansion; however, they only expand the Vγ9Vδ2 cell subset and are ineffective for Vδ1 T cells, resulting in cell waste and an inability to utilize the unique capabilities of Vδ1 T cells. Waller found that treating PBMCs with an anti-CD2 monoclonal antibody could produce an IL-12-dependent signal that not only protected γδT cells from mitogen-induced apoptosis but also promoted cell proliferation with no negative effect on the function of amplified γδT cells [393]. Foster et al. stimulated PBMCs with ConA and observed a 2846-fold expansion of γδT cells after 19 days [394]. Cooper et al. developed a method to expand polyclonal CAR-γδT cells by electroporating PBMCs with the sleeping beauty (SB) transposon system and then sorted the cells to obtain CAR-γδT cells containing a broad combination of Vγ and Vδ chains. When co-cultured with γ-irradiated, CD19^+^ K562-derived artificial antigen-presenting cells in the presence of soluble IL-2 and IL-21, these sorted anti-CD19 CAR-γδT cells expanded considerably, yielding a substantial number of polyclonal CAR-γδT cells with a memory phenotype and remarkable specific anti-tumor activity [395]. These approaches may address the problem of insufficient cell supply for clinical CAR-γδT therapy, but their feasibility has yet to be tested in the clinic.

2. Inadequate CAR optimization for γδT cells

As γδT cells differ significantly from αβT cells, it is important to construct appropriate CAR molecules that consider the characteristics of γδT cells for improved performance. Although γδT cell therapy has demonstrated good safety in recent studies, the likelihood of CAR-related on-target/off-tumor toxicity cannot be neglected. The ability of γδT cells to recognize antigens in a non-MHC-restricted manner gives them an advantage in tackling this challenge. Considering the preexisting capacity of γδTCR in tumor antigen recognition and signal transduction, Anderson et al. constructed a CAR composed of an anti-GD2 scFv linked with a DAP10 endodomain that enabled costimulation to supplement the endogenous γδTCR signal [396]. After being expressed in Vγ9Vδ2 T cells, this costimulation-only CAR recognized GD2 and then transmitted DAP10 signals in concert with Vγ9Vδ2 TCR signaling to fully activate the CAR-γδT cells. Moreover, by building an effective “AND” logic gate with the inherent γδTCR, this novel CAR offers γδT cells a more precise tumor-killing activity, thus minimizing on-target/off-tumor toxicity [396]. The DAP10 signal typically provided by NKG2D is essential for γδT cell activation, yet most tumor cells may block this signal by decreasing and/or shedding NKG2D ligands, resulting in tumor immune evasion [397]. Therefore, this new CAR may also prevent tumor cells from evading γδT cell attack when appropriate antigen recognition components are used. As mentioned earlier, exhaustion is a serious challenge that negatively impacts the efficacy of all T cell therapies. However, it can be alleviated by modifying the CAR structure in CAR-γδT cell therapy. Anderson and Pe'er found that typical CD3ζ-carrying CARs triggered tonic signaling network activation in both αβT and γδT cells, which resulted in T cell exhaustion. An alternative CAR that lacked CD3ζ but contained DAP10 stimulatory domains did not cause tonic signaling but efficiently activated γδT cells in the presence of CAR-specific stimuli or cognate leukemic cells [398]. These findings indicate that altering CAR design could mitigate engineered γδT cell exhaustion and on-target/off-target cytotoxicity, two factors that consistently affect the clinical efficacy of CAR-T cells [399].

The improvement and maintenance of in vivo persistence in γδT cells also represent obstacles that may be overcome by employing molecular factors that support cell survival and functionality. Consequently, it is beneficial to add more appropriate costimulators to CAR for γδT cells. Although it has been demonstrated that the most widely used costimulatory molecules in CAR-T therapy, CD28 and 4-1BB, are expressed in γδT cells and control their proliferation and activation [400, 401], it is unclear how these molecules affect the fate of γδT cells when working synergistically with other CAR components, particularly in the case of CD28, whose role in γδT cells remains controversial [402]. Other favorable γδT cell factors such as CD27, which has been demonstrated to increase γδT cell proliferation and activation [403], are also worth investigating and potentially incorporating into CARs.

Despite significant progress in preclinical and early clinical studies with CAR-γδT cells in hematological malignancies, their clinical efficacy in solid tumors remains uncertain and may be affected by the complexity and heterogeneity of the immunosuppressive TME [386]. To address the existing and forthcoming challenges in clinical applications, a thorough understanding of CAR-γδT cells is required. However, the potential of CAR-γδT cell therapy cannot be overlooked. The future development of CAR-γδT cell therapy still deserves considerable attention.

### CAR-NKT: new treatment options for solid tumors

NKT cells are a subset of innate T cells that acquire the properties of NK cells after developing in the thymus. Therefore, NKT cells express both NK cell and T cell receptors and possess both specific and non-specific killing functions. They also have the capacity to modulate recruitment, activation, and immune response in numerous innate and adaptive immune cells by secreting various cytokines and chemokines or through direct interaction. There are two types of NKT cells, with type I (invariant natural killer T, iNKT) receiving the majority of research attention. Studies have found that NKT cells play an important role in various diseases, such as cancers, infections, and autoimmune diseases [404]. Therefore, adoptive therapy based on NKT cells has been explored in clinical research [405–407]. In contrast to NK cells, the direct killing action of NKT cells depends on the presentation of CD1d glycolipids to invariant TCR, similar to how T cells recognize HLA-restricted targets. However, because most tumors are CD1d-negative, NKT cells are unable to perform the direct killing function of NK cells; however, the use of CAR can help compensate for this deficiency of NKT cells.

Considering the success of CAR-T cell therapy, CAR has also been investigated in relation to NKT cells. Using a retrovirus, Metelitsa et al. constructed PBMC-derived CAR-NKT cells that target GD2 and CD19 and discovered that they could destroy tumor cells both in vitro and in vivo. They also found that CAR can induce Th1-like polarization of NKT cells when 4-1BB serves as the costimulatory molecule [408, 409]. Karadimitris et al. developed lentivirus-based, CD19-targeting CAR-NKT cells and demonstrated their CAR- and CD1d-dependent anti-tumor effects in vitro and in vivo [410]. Uslu et al. used mRNA electroporation to construct PBMC-derived CAR-NKT cells that targeted CSPG4 and found that the anti-tumor effect of CAR-NKT cells was comparable to that of CAR-T cells but showed better in vivo safety [411]. These studies support the feasibility of developing CAR-NKT cell therapies.

#### Advantages

CAR-NKT cell therapy may compensate for the shortcomings of CAR-T cell therapy owing to its advantage of dual NK cell and T cell properties.

1. Safety

In contrast to conventional CAR-T cell therapy, CAR-NKT cell therapy typically does not result in an excessive immune response. Uslu et al. found that CSPG4-targeting CAR-NKT cells produced noticeably fewer CRS-related cytokines (such as IL-6) while exhibiting tumor cytotoxicity comparable to that of CAR-T cells [411]. Furthermore, CAR-NKT therapy has not led to severe CRS or NT in conducted clinical trials.

2. Multiple killing modes

Owing to their NK-like properties, CAR-NKT cell exhibits diverse tumor-eliminating methods (Fig. 7B). In addition to the targeted killing function mediated by CAR, CAR-NKT cells can also directly kill tumor cells in a target-independent manner by utilizing FASL, TNF-α, TRAIL, and perforin/granzyme, which makes them preferable for eliminating extremely heterogeneous tumors. As the non-specific killing of NKT cells is dependent on the expression of CD1d by target cells, NKT is not effective against CD1d-negative tumor cells; however, combining it with CAR can compensate for this limitation. According to studies by Metelitsa and Karadimitris, CAR-NKT cells can possess a dual-specific killing ability via CAR- and CD1d-dependent mechanisms [408, 410], yielding a stronger anti-tumor effect than CAR-T cells and effectively eliminating relapsed lymphoma. Moreover, CAR-NKT cells eradicated brain tumors when CAR-T cells could not, suggesting that CAR-NKT cells can cross the blood–brain barrier [410]. These findings demonstrate that CAR-NKT cells might be more effective against tumors than CAR-T cells.

3. TME improvement

CAR-NKT cells can recruit and activate other immune cells by initiating the Th1/Th2 cytokine cascade, which includes activating NK cells and maturing DCs to stimulate cytotoxic T lymphocytes and inhibit TAMs. Metelitsa et al. discovered that CAR-NKT cells that used 4-1BB as a costimulatory molecule tended to polarize toward Th1 cells, with increased levels of both IFN-γ and GM-CSF but a lack of IL-4 and IL-10. Furthermore, they found that CAR-NKT cells can also kill CD1d^+^ M2 macrophages [408], indicating their potential ability to improve the TME.

4. Universal therapy

In contrast to T cells, NKT cells recognize CD1d, a non-classical MHC-I-like molecule. Consequently, CAR-NKT cell therapy does not induce GvHD, making universal CAR-NKT cell therapy more feasible. According to the current anti-CD19 CAR-NKT phase 1 clinical trial (NCT03774654) data published by Athenex Inc., seven patients who received allogeneic CAR-NKT therapy exhibited a good safety profile and did not develop GvHD [412, 413]. This demonstrates the potential of CAR-NKT cell therapy as a universal therapy.

#### Research progress

Thus far, only five CAR-NKT clinical trials have been registered, four of which are ongoing (Fig. 2A, Table 2). Kuur Therapeutics published the interim phase 1 clinical trial results of their autologous GD2-targeting CAR-NKT cells for the treatment of relapsed or resistant neuroblastoma (NCT03294954) in 2020. In this study, CAR-NKT cells were produced from the PBMCs of three patients via retroviral transduction and were expanded for 15, 14, and 9 days in the case of patients 1, 2, and 3, respectively. After being infused into patients, CAR-NKT cells secreting IL-15 expanded in vivo, effectively infiltrated tumor sites, and mediated tumor regression. One of the three patients achieved CR and bone metastases regressed, and none of the patients experienced significant side effects [414]. This study suggests that NKT cells, despite being rare in PB, can be genetically modified to express a CAR, expanded on a clinical scale, and safely used to treat patients. Furthermore, three clinical trials of CD19-targeting CAR-NKT cells (NCT03774654, NCT05487651, NCT04814004) for the treatment of B cell lymphoma are ongoing (Table 2), but no data have been published yet.

#### Challenges and potential solutions

1. Cell scarcity

The main problem with CAR-NKT cell therapy is the scarcity of NKT cells, which only account for 0.1–1% of PB T lymphocytes. While CAR-NKT cells can be expanded in vitro to a sufficient quantity and then reinfused back into the body, this long-term expansion process may also reduce their potency. In a GD2-targeting CAR-NKT cell clinical trial, CAR-NKT cells with longer ex vivo expansion periods exhibited worse persistence when reinfused into patients. Additionally, NKT cells gradually lose their function when they are repeatedly stimulated in vitro with tumor cells [414]. This implies that although NKT cells can proliferate substantially in vitro and generate a sufficient number of cells for use in therapy, their activity might be compromised during prolonged ex vivo expansion.

Numerous attempts have been made to address this issue, and several potential solutions have been proposed. In 2019, Yang et al. genetically engineered iNKT TCR into hematopoietic stem cells (HSCs) to generate engineered iNKT cells. These HSC-iNKT cells were very similar to endogenous iNKT cells and effectively inhibited the growth of both multiple myeloma and melanoma xenografts in vivo [415]. Fujii et al. attempted to obtain a large number of iNKT cells using iPSCs by creating iPSCs from NKT cells isolated from PB and then converted these iPSCs back to iNKTs after the iPSCs were expanded. These iPSC-derived NKT cells were significantly more potent than parental iNKTs in terms of cytokine secretion and cytotoxicity [416]. Currently, these iPSC-derived iNKTs are being tested in clinical trials for head and neck cancer in Japan [417].

2. Poor persistence in vivo

The activity and proliferation of CAR-NKT cells gradually decline after being infused into the body, which is frequently associated with tumor recurrence. Consequently, multiple infusions of CAR-NKT cells are required to achieve the desired therapeutic effect, which exacerbates the problem of NKT cell scarcity. Attempts to improve the in vivo persistence of CAR-NKT cells, rather than repeated injections, represent a more effective strategy to address their limitations to clinical application. Metelitsa et al. showed that CD62L^+^ NKT cells outperform CD62L^−^ NKT cells in terms of proliferation and survival after repeated antigen stimulation, and that CD62L^+^ NKT cells have better persistence and anti-tumor activity in mouse tumor models [409]. Consequently, they investigated strategies to enrich CD62L^+^ NKT cells and discovered that introducing certain cytokines would produce the desired outcomes. In preclinical and clinical trials of anti-GD2 CAR-NKT cells, they found that IL-15 co-expression increased the proportion of CD62L^+^ cells and improved CAR-NKT cell persistence in vivo [414, 418]. IL-21 is another cytokine that is specifically protective of CD62L^+^ NKT cells and boosts their effector capabilities and can be used to expand CAR-NKT cells ex vivo and improve therapeutic efficacy [419]. These results imply that it is feasible to produce CD62L-enriched NKT cells for efficient cancer immunotherapy.

In conclusion, even though few studies have been completed thus far, the existing results indicate that CAR-NKT cell therapy has considerable potential for the treatment of solid tumors, particularly when its limitations are addressed.

## Gene transduction strategies in ACT

**Fig. 8:**
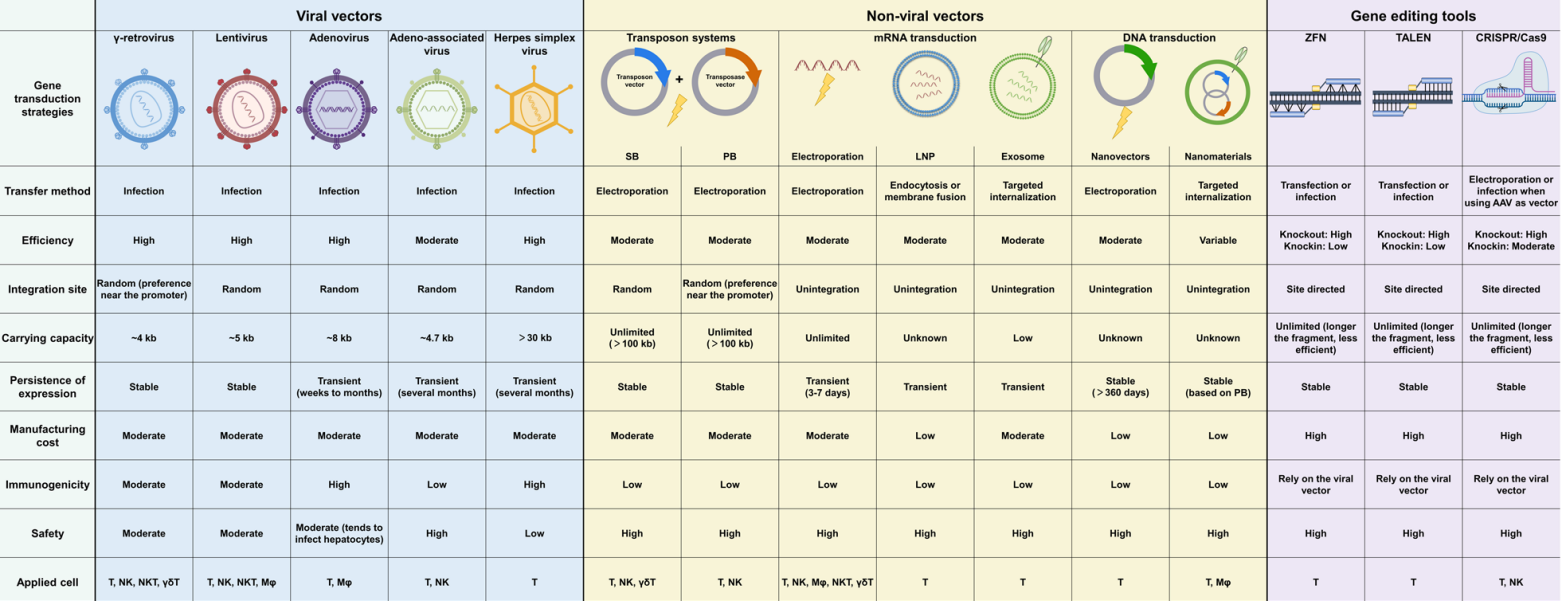
Summary of gene transduction strategies in current adoptive cell therapies. Mφ, macrophage; SB, sleeping beauty transposon system; PB, piggyBac transposon system; LNP, lipid nanoparticle; ZFN, zinc finger nuclease; TALEN, transcription activator-like effector nuclease; and CRISPR/Cas9, clustered regularly interspaced short palindromic repeats-associated protein 9

ACT requires the introduction of CAR or TCR genes into recipient cells as well as the precise control of gene expression. The emergence of a series of gene transduction strategies has advanced the development of ACT. An ideal gene transduction strategy should be safe, have low immunogenicity, and be able to carry as large a fragment of the gene as feasible while also allowing the transduced gene to remain stable or be expressed long term in the recipient cells. Currently, three types of gene transduction strategies are frequently used in ACT: (1) viral vectors, including retrovirus, lentivirus, and adenovirus vectors; (2) non-viral vectors such as transposons, mRNA transduction, and DNA transduction; and (3) gene editing tools such as CRISPR/Cas9, TALEN, and ZFN (Fig. 8). Although these gene transduction strategies have considerably enhanced the potential of engineered immune cells, they are limited by problems such as insertion mutation, poor transduction effectiveness, and high cost.

### Viral vectors

#### γ-retrovirus

The retroviral family contains seven members, among which only γ-retrovirus is clinically used. All retroviruses have three structural genes: *gag*, which encodes viral structural proteins; *pol*, which encodes reverse transcriptase and integrase for viral replication; and *env3*, which encodes viral envelope glycoproteins. The γ-retrovirus can transport a sizable amount of genetic cargo, transduce various cell types, integrate foreign genes into the host’s genome to ensure sustained expression, and scale up production rapidly for clinical translation. Owing to these advantages, γ-retrovirus was the first viral vector approved for use in clinical trials [420], as well as the first vector employed in CAR-T cell preparation [421]. In 2003, Brentjens et al. successfully harvested 51% positively transduced CAR-T cells by infecting T cells with γ-retrovirus carrying anti-CD19 CAR genes. These CAR-T cells effectively lysed CD19-positive tumor cells and prolonged the survival time of tumor-bearing mice. The γ-retrovirus has been extensively used in clinical trials of immune cell therapy and has resulted in positive outcomes. Davila et al. used γ-retrovirus to produce CD19-targeting CAR-T cells, which, when combined with chemotherapy in the treatment of 16 patients with B cell acute lymphoblastic leukemia, resulted in a CR as high as 88% [422]. Liu et al. used γ-retrovirus to construct CAR-NK cells expressing anti-CD19 CAR, IL-15, and inducible caspase-9 with a final transduction efficiency of 49.0% (range 22.7–66.5%). These CAR-NK cells were tested in a clinical trial involving 11 patients with CD19-positive tumors, with eight responding and seven achieving CR [319]. Furthermore, Yescarta and Tecartus, two currently marketed CAR-T cell therapy products, both use γ-retrovirus as the gene delivery vector, demonstrating the applicability of γ-retrovirus in the preparation of cell therapy products. However, the integration site of γ-retrovirus is often close to the promoter, which can alter the activity of nearby genes. Therefore, γ-retroviruses have the potential to transform healthy cells into cancerous cells if integration occurs in proto- or anti-oncogenes [354, 423]. Some patients in a gene therapy clinical trial with γ-retrovirus experienced an insertion mutation, leading to the occurrence of clonal T cell acute lymphoblastic leukemia [424]. However, excessive concern is unwarranted because it is unlikely to occur frequently. Research has shown that mature T cells can resist tumorigenic transformation via apoptosis and epigenetic mechanisms [425]. Furthermore, no evidence of vector-induced cell immortality was found in a long-term trial (> 500 patient-years of follow-up) involving the use of γ-retrovirus to transduce T cells to treat HIV [426]. Moreover, if necessary, adding suicide elements to CAR could eliminate the potential transform risk caused by γ-retrovirus integration. Nevertheless, owing to the drawbacks of γ-retroviruses, such as their inability to infect non-dividing cells and the greater influence on the host transcriptome [427], lentivirus vectors have gradually replaced them in clinical trials [428].

#### Lentivirus

The lentiviral vector was established by transforming human immunodeficiency virus-1 (HIV-1). Lentiviruses have a unique cis-acting element called central polypurine tract (cPPT) that allows them to efficiently transduce resting T cells without undergoing mitosis. The lentiviral genome includes *gag*, *pol*, and *env,* which are shared by retroviruses, as well as two regulatory genes, *tat* and *rev*, and four accessory genes, *vif*, *vpr*, *vpu*, and *nef*, which are responsible for coding essential proteins for virus replication, binding, infection, and release [429]. The lentiviral vector has been updated and iterated to the third generation. Additionally, its safety was increased by incorporating self-inactivating transformations, reducing homologous sequences, deleting non-essential genes, dispersing viral genes in different plasmids, and other methods [430–432]. Only the *gag*, *pol*, and *rev* genes from the HIV-1 genome are retained by the packaging system of the third-generation lentiviral vector, and these genes are dispersed across three separate plasmids (pGag/Pol, pRev, pVSV-G). Furthermore, the lentiviral LTR sequences in the plasmid-encoding gene of interest are altered to be self-inactivating to further improve safety by preventing recombination. To improve target gene expression, the woodchuck hepatitis virus post-transcriptional regulation element (WPRE) was introduced into the 3′LTR to stabilize transcripts, which can quadruple the expression of the target gene when combined with cPPT.

Compared with γ-retroviral vectors, lentiviral vectors have a broader range of applications, higher transduction efficiency, larger transgene load, and lower immunogenicity. Kymriah, Breyanzi, Abecma, Carteyva, Carvykti, and CT103A are commercial CAR-T cell products that all use lentiviral vectors to introduce CAR genes. Furthermore, lentiviral vectors are also being used in an increasing number of clinical trials for cell therapy, which have shown promising results. In a study by Maude et al., 30 patients with r/r acute lymphoblastic leukemia were treated with lentivirus-transduced second-generation CAR-T cells. Consequently, 90% of these patients experienced CR, and the 6-month event-free survival rate was 67%, while the 2-year overall survival rate was 78% [329]. Using lentiviral vectors, Wang et al. produced anti-CD19 CAR-T cells by infecting CD4^+^ and CD8^+^ Tcm subsets. These T cell products were safely administered to eight patients with B cell non-Hodgkin lymphoma, six of whom were progression free at 1 year [433].

However, lentiviral vectors have some limitations. The risk of insertion mutation, for instance, is inevitable because the integration site of lentiviral vectors is still random. There have been instances of T cell clonal expansion due to CAR insertion into TET2 [268] and CBL [434] genes during clinical treatment. Furthermore, the multi-plasmid co-transformation system used in the lentiviral packaging process makes acquiring lentiviruses difficult, expensive, and prone to inconsistencies in quality between batches [435]. Finally, lentiviruses typically have a very low infection efficiency for certain cells, such as NK cells. These issues have motivated researchers to continue exploring more effective gene transfer techniques.

#### Other viral vectors

In addition to the retroviral family, other viruses are also used for gene transduction, such as adeno-associated virus, adenovirus, and herpes simplex virus. Adeno-associated viruses can infect both dividing and non-dividing cells and are generally not pathogenic or cytotoxic. The risk of insertion mutations is lower with adeno-associated viruses than with retrovirus-derived vectors because of their low rate of gene integration. However, they are greatly limited by their limited packaging capacity (< 5 kb). Although adenoviruses, which have a packaging size of up to 8 kb, can also transduce both dividing and non-dividing cells with a low risk of insertion mutation, they are easily rejected by the host innate immune response [436]. Herpes simplex virus is a large-capacity vector (> 30 kb) with multiple foreign gene insertion sites and high efficiency of gene transduction; however, it exhibits limitations regarding immunogenicity and poor targeting, and the transgene cannot be expressed for a long time in certain organs, such as the brain.

### Non-viral vectors

#### Transposon

Transposons are natural and mobile DNA segments that can change their position within the genome [437]. Transposon systems comprise a transposase and target genes with transposase binding sites. The target gene is cut off and moved by the transposase in conjunction with the inverted terminal repeats at both ends of the target gene [438]. The transposon system has been used extensively in academic research and clinical trials because of its advantages of large gene load, high transduction efficiency, ease of use, limited immunogenicity, and low industrial cost [439]. The two most popular transposon systems are sleeping beauty (SB) and piggyBac (PB), both of which are composed of two plasmids that express the transposase and carry the target gene. Transposition efficiency is closely related to transposase activity. Through the modification of terminal repeats and transposase, transposition systems with different DNA cleavage and transposition activities have been developed, such as SB11 and SB100X of the SB system, and pB and 7pB of the PB system [440–442].

The transposon system is an efficient and flexible technique for genetically altering T cells for cancer therapy. SB11 transposase has been used in clinical investigations. In a phase 1/2 clinical trial, Singh et al. produced CD19-targeting CAR-T cells with a CAR-positive rate of over 90% using the SB11 transposon, and these cells demonstrated powerful anti-tumor effects [443] However, when Jin et al. compared the transposition activity of SB11 and SB100X, they discovered that SB100X was roughly 3.6 times more effective than SB11 at producing CAR-T cells, indicating that the potential of SB system in T cell editing has yet to be fully explored [444]. Moreover, the SB100X transposon can successfully transfer multiple genes into human T cells using a single electroporation procedure [445], which may have unparalleled advantages for the creation of multi-target CAR-T cells. Additionally, SB transposons have been used in the development of TCR-T cells. Deniger et al. used the SB11 transposon to construct ERBB2 mutation-specific TCR-T cells and demonstrated their potent response to tumor cells and considerable in vitro lethality [446].

Another promising tool for engineering T cells is the PB transposon system. Nakazawa et al. used the PB transposon to introduce eGFP gene into T cells and observed sustained transgene expression for over 6 months in PB-modified T cells [447]. Considering this, the use of the PB transposon system to produce CAR-T cells has recently grown in popularity. For instance, Kubo et al. constructed EPHB4-targeting CAR-T cells with PB and achieved a CAR-positive rate of 78.5%. These cells also exhibited notable memory and low exhaustion characteristics [448]. In a phase 1 clinical trial for multiple myeloma, anti-BCMA CAR-T cells produced using the PB system also showed positive outcomes and had fewer side effects than other comparable anti-BCMA CAR-T cell products [449].

However, the use of transposon systems also faces some challenges that need to be resolved. First, the size of the target gene has a substantial impact on the transposition efficiency of SB transposons; the transposition efficiency is significantly reduced when the target gene is larger than 5 kb. Second, transposon systems that rely on electron transduction consistently result in a large number of cell deaths and are prone to the integrating multiple copies of the target gene [444]. Finally, because SB transposon prefers to be integrated into transcriptional units, and the PB transposon tends to integrate at the transcription start sites and CpG islands [437, 450], both systems face the risk of insertion mutation or even carcinogenesis. In a genome-wide mapping study, the PB system was demonstrated to integrate target genes into 888 known proto-oncogenes in T cells [450]. Additionally, transposons naturally allow transduced genes to repeatedly change their genomic positions, which may result in unpredictable outcomes [451]. It may be possible to increase the safety of the transposon system by shielding transcriptional regulatory elements to prevent the influence on genes close to the integration site or by modifying the transposase to increase the specificity of target recognition, but these measures have not yet been tested clinically [452]. It remains to be further investigated whether the transposon system is superior to other gene transduction methods, particularly in terms of safety.

#### mRNA transduction

1. mRNA electroporation

mRNA electroporation is a technique that modifies mRNA to increase its stability before introducing it into the cytoplasm via electron transduction for expression. Gene transduction in this manner challenges the long-held assumption that that mRNA is unstable and cannot be used as a medicinal molecule. Electroporated mRNA is immediately available in the cytosol and does not need to enter the nucleus, which not only improves expression efficiency but also eliminates the possibility of insertional mutagenesis. Therefore, mRNA electroporation may be one of the most secure methods of effective gene transduction. Furthermore, mRNA electroporation also offers advantages such as high transduction efficiency, greater versatility (can transduce nearly any cell type, including quiescent or slowly proliferating cells as well as primary immune cells), easy design and optimization, and the ability to rapidly produce desired cells at a lower cost [453]. Consequently, studies involving mRNA electroporation are becoming more frequent.

Beatty et al. electroporated mRNA encoding mesothelin-specific CAR into activated T cells and successfully produced clinical-grade CAR-T cells with over 91% mean cell survival and a CAR-positive rate of over 98%. These CAR-T cells demonstrated substantial anti-tumor activity in patients without overt evidence of off-tumor/on-target toxicity [454]. These findings support the idea that adoptive transfer of mRNA-transduced CAR-T cells is feasible and safe. Although the currently preferred methods for CAR-T cell preparation are based on retroviral or lentiviral transductions, 10% of patients die from their disease progressing before receiving treatment because of the laborious manufacturing process [455]. mRNA electroporation technology can greatly reduce the preparation time for clinical-grade CAR-T cells and has therefore attracted considerable attention as it would enable patients with aggressive disease to receive CAR-T therapy within a short period. Krug et al. used mRNA electroporation technology to produce a sufficient number of CEA-specific CAR-T cells in only 10 days, resulting in 25.7 ± 2.9% CAR-positive T cells after electroporation and cryoconservation [456]. This reflects the major advantages of mRNA electroporation in clinical applications.

Limited by the inherent instability of mRNA molecules, mRNA electroporation typically only causes transient expression of CAR. This problem can be remedied by repeated injections of mRNA-transduced CAR-T cells in patients, but this increases treatment expenses. However, this deficit of mRNA-transduced CAR-T cells is an advantage in terms of safety because it not only eliminates the risk of insertion mutations but might also avoid unpredictable repercussions such as cross-reaction with normal tissues due to CAR-constitutive expression [191]. Furthermore, electroporation usually causes irreversible damage to cells, which limits the application of mRNA electroporation technology. Therefore, researchers are exploring other alternatives.

2. Lipid nanoparticle-mediated mRNA transduction

In recent years, nanomaterials have also been used in ACT. Among them, lipid nanoparticles (LNPs) have achieved promising results in delivering mRNA to produce CAR-T cells. LNPs enter cells and deliver mRNA through endocytosis or membrane fusion. While maintaining transduction efficiency similar to that of electroporation, LNP exhibits low cytotoxicity [457, 458]. Mitchell et al. successfully generated LNP-derived CAR-T cells and displayed in vitro anti-tumor activity comparable to that of electroporation-derived CAR-T cells [457, 458]. However, LNP-mediated mRNA transduction also faces issues such as poor stability, low biocompatibility, poor degradability, and induction of side effects in vivo [459]. Therefore, researchers continue to search for new strategies to address these limitations.

3. Exosome-mediated mRNA transduction

Exosomes secreted by living cells contain many functional molecules that can mediate material transfer and communication between cells through corresponding receptors. Exosomes have some unique advantages, such as structural stability, effective protection of mRNA from degradation, low immunogenicity, ability to cross the blood–brain barrier, and ease of engineering [459, 460]. Therefore, researchers have attempted to generate CAR-T cells via exosome-mediated gene transduction [459]. Loading CAR-encoding mRNA and expressing anti-CD3/CD28 scFvs on the membrane allow exosomes to be directly used for the activation of and precise delivery of CAR-encoding mRNA to T cells to generate CAR-T cells with tumor-killing functions [459]. However, the cytotoxicity to target cells of exosome-generated CAR-T cells is slightly weaker than that of lentiviral-generated CAR-T cells, which may be due to the low expression level of CAR mediated by exosomes. Furthermore, although the engineered exosomes have not yet been tested in vivo, they provide a potential new strategy for the in vivo preparation of CAR-T cells and require further optimization.

#### DNA transduction

Similar to mRNA transduction, the delivery of CAR-encoding DNA to immune cells has also been attempted in ACT. DNA has the advantage of being more stable than mRNA.

1. DNA nanovector electroporation

Harbottle et al. produced a novel DNA nanovector called nS/MARt through several modifications of the original plasmid pEPI [461]. The gene encoding CAR can be loaded on the vector and transduced into cells by electroporation, which can mediate long-term stable expression of the target gene. Compared with lentivirus vector transduction, nS/MART-mediated gene transduction had similar transduction efficiency but achieved higher CAR expression levels. In terms of function, CAR-T cells produced by nS/MARt showed stronger infiltration ability and tumor-specific lysis in vivo. Notably, nS/MARt-generated CAR-T cells induced gene expression for at least 360 days in Jurkat76 cells by electroporation, reflecting the characteristics of stable gene expression mediated by a viral vector but without the risk of genome integration and insertion mutation [461]. To translate nS/MARt-generated CAR-T cells to clinical applications, researchers also devised a production procedure that could provide clinical-grade CAR-T cells capable of transforming from an initial 1 × 10^9^ T cells to 3.6 × 10^8^ functional CAR-T cells within 5 days. This considerably accelerated the generation of CAR-T cells, as a period of 12–14 days is necessary to produce a sufficient CAR-positive cell quantity using conventional lentiviral vectors [462]. Although this nanovector has yet to be tested in clinical trials, it is expected to have a profound impact on the application of CAR-T cell therapy.

2. Nanomaterial-mediated targeted DNA delivery

A faster approach for generating CAR-T cells is to manufacture immune cells directly in vivo. To bypass the laborious and expensive process of autologous CAR-T cell production in vitro, Stephan et al. employed DNA-carrying nanoparticles to directly program host T cells with leukemia-specific CAR genes in vivo [463]. These nanoparticle-programmed CAR-T cells can rapidly eliminate cancer cells and alleviate tumor burden in mice with B cell acute lymphoblastic leukemia. Kim et al. used intratumoral injection of nanocomplexes to deliver IFN-γ-secreting CAR encoding plasmids to macrophages in mice with Neuro-2a xenografts, generating high amounts of CAR-M cells in tumors (accounting for 82% of CAR-positive cells). With the help of autocrine IFN-γ, these cells polarized from the M2 to the M1 phenotype and inhibited tumor growth by directly phagocytosing tumor cells and stimulating anti-tumor responses [375].

With the recent FDA approval of Onpattro from Alnylam, a medication that encapsulates therapeutic siRNAs into an LNP, clinical acceptance of the nanoparticle delivery system has advanced. Nanomaterials have considerable potential for use in CAR-T cell preparation or as an auxiliary strategy to deliver necessary components for transposon and gene editing systems.

### Gene editing tools

#### CRISPR/Cas9

Currently, lentiviral and retroviral vectors are the two main tools for gene transduction in cell therapy. However, both of these vectors can randomly integrate into problematic regions of the genome, thus compromising the quality and therapeutic effects of engineered cells [464, 465]. Therefore, the CRISPR/Cas9 technology, which enables precise gene editing while reducing the possibility of random insertion, has sparked widespread interest.

The CRISPR/Cas9 system is an RNA-guided, targeted gene editing technology that mainly comprises two components: guide RNA (gRNA), which can recognize certain DNA sequences, and Cas9 endonuclease, which cuts DNA at the target sites. Under the guidance of gRNA, Cas9 can localize and cut DNA precisely at the target site to produce double-strand breaks (DSBs), which subsequently activate two distinct repair mechanisms: non-homologous end joining (NHEJ) and homology-directed repair (HDR). NHEJ can directly connect the break sequences when templates are absent and cause insertion/deletion mutations, whereas the HDR pathway is activated to produce specific insertions, deletions, or mutations in the presence of a donor template [466]. In addition to its capacity for precise gene editing, the CRISPR/Cas9 system has several advantages, including easy design, quick implementation, low cost, and excellent scalability [467]. Because of this, the application of CRISPR/Cas9 system has expanded to almost all genomic targets and has also accelerated the development of engineered cell therapy.

The CRISPR/Cas9 system has demonstrated potential for use in CAR-T cell and TCR-T cell therapies. As it enables simultaneous gene editing at multiple loci, CRISPR/Cas9 is regarded as a groundbreaking gene editing tool for producing universal CAR-T cells. In 2017, Liu et al. [468] used CRISPR/Cas9 technology to knock out *TRCA* (encoding the endogenous TCRα subunit) and *B2M* (encoding an essential subunit of the MHC-I molecule) in T cells, producing universal CAR-T cells that greatly reduced tumor growth in mouse models. Since then, an increasing number of studies has been conducted and several have recently started clinical trials [469]. The CRISPR/Cas9 system can also be used to enhance the function of CAR-T cells by deleting immunosuppressive genes or introducing CAR-expressing segments into particular loci. Zhang et al. [470] used CRISPR/Cas9 to construct lymphocyte activation gene-3 (LAG-3)-deficient CAR-T cells and discovered that these cells had potent anti-tumor activity in both in vitro and in vivo settings. Ren et al. [218] employed CRISPR/Cas9-mediated multi-gene editing technology to produce CAR-T cells devoid of TCR, MHC-I, and PD-1. These CAR-T cells exhibited reduced allogenic reactivity and elevated anti-tumor activity in vivo. Eyquem et al. [172] employed CRISPR/Cas9 technology to direct a CD19-specific CAR coding sequence into the *TRCA* locus, placing it under the regulation of endogenous regulatory elements. This not only led to homogeneous CAR expression but also enhanced T cell potency by averting CAR tonic signaling, establishing effective internalization and re-expression of the CAR following single or repeated exposure to an antigen, and delaying effector T cell differentiation and exhaustion. Finally, CRISPR/Cas9 technology also offers a solution to the issue of endogenous and exogenous TCR potentially competing and forming a mixed dimer in TCR-T cell therapy. When Legut et al. [471] used CRISPR/Cas9 technology to remove the natural TCR from T cells and introduce a cancer-reactive TCR, the surface expression of transgenic TCR and the sensitivity of TCR-T cells to the antigen were both markedly improved.

However, the CRISPR/Cas9 system, which uses NHEJ- and/or HDR-mediated gene integration, is not without shortcomings. HDR-mediated gene integration occurs infrequently, and the insertion of large gene fragments is necessary for engineered cell therapy, which makes it more challenging to acquire sufficient edited cells. Although the frequency of DNA integration caused by NHEJ is over 1000 times higher than that of HDR [472], NHEJ-mediated DSB repair is prone to frameshift mutations after insertion/deletion, leading to the interruption of gene open reading frames and premature termination [473]. Furthermore, it was shown that CRISPR/Cas9-mediated gene editing has potential off-target effects, in which DNA double-stranded cleavage occurs outside the target site and causes random insertion/deletion mutations by activating cell NHEJ repair mechanisms [474, 475]. Finally, CRISPR/Cas9-mediated multiple gene editing for immune cells, which requires the simultaneous introduction of multiple genes encoding Cas9 and gRNAs, typically produces multiple cell populations with different combinations of knockout phenotypes. For example, Moriarity et al. used the base editor BE4 for mRNA-mediated editing to knockout *TRAC*, *PDCD1*, and *B2M* in T cells and generated only 21.9 ± 1.1% triple knockout cells, accompanied by a large ratio of single and double knockout cells. After optimizing the system, the proportion of triple knockout cells was increased to more than 80% and generated anti-CD19 CAR-T cells [476]. Collectively, these existing technical imperfections restrict the widespread use of CRISPR/Cas9 technology in immune cell therapy. However, improvements in the delivery efficiency and editing precision of Cas9 can lead to major advancements in CRISPR/Cas9 technology in the future.

#### TALEN

In addition to the CRISPR/Cas9 system, TALENs and ZFNs can also precisely edit genes. Although both have been used in cell therapy, their primary application is to knock out TCR, MHC, or CD52 genes in T cells to generate universal CAR-T cells.

TALEN is an artificially modified restriction endonuclease that consists of an N-terminal domain containing a nuclear localization signal, a central domain with a typical tandem TALE repeat sequence that can recognize a specific DNA sequence, and a C-terminal domain with a FokI endonuclease. Through the DNA recognition module, TALEN binds to the target DNA site, uses FokI nuclease to create a DSB, and, similar to CRISPR/Cas9, uses the inherent HDR or NHEJ repair process to insert or delete specific sequences.

As mentioned previously, TALEN technology has been used to generate universal CAR-T cells. After introducing CD19-specific CAR into T cells using lentiviral vectors, Qasim et al. used TALEN to knock out *TRAC* and *CD52* and construct universal CAR-T cells (UCART19) [477]. When these UCART19 cells were infused into two infants with r/r CD19^+^ B cell acute lymphoblastic leukemia, molecular remissions were achieved within 28 days, and UCART19 cells persisted during the course of therapy. Moreover, Jo et al. simultaneously disrupted and repurposed the endogenous *TRAC* and *B2M* loci using TALEN-mediated gene editing and AAV6-dependent gene insertion to produce TCR- and HLA-ABC-deficient T cells expressing CAR and the NK-inhibitor HLA-E. These hypoimmunogenic universal CAR-T cells blocked the GvHD response and avoided being destroyed by NK and alloresponsive T cells, prolonging their anti-tumor activity [478]. These investigations show the potential of TALEN for immune cell therapy. Notably, the editing effectiveness of TALEN is comparable to, or even superior to, that of CRISPR/Cas9. Jain et al. [479] reported that TALEN demonstrated comparable or even superior editing effectiveness to Cas9 in the heterochromatin region. In some cases, the editing efficiency of TALEN can exceed five times that of the CRISPR/Cas9 system. Cas9 appears to be more effective in editing euchromatin sites with high transcriptional activity in the genome. This suggests that TALEN may be a superior option for some difficult-to-edit gene regions, and researchers can choose a suitable gene editing method according to the target site.

#### ZFN

ZFN is a fusion protein composed of zinc lipoprotein that specifically recognizes and binds to DNA and a FokI endonuclease domain. Similar to TALEN, the ZFN system can also mediate specific DSBs of target genes, which then can be repaired through NHEJ or the HDR pathway, resulting in gene knockout or insertion [480]. ZFN has been successfully used in allogeneic CAR-T cell preparation. As early as 10 years ago, Torikai et al. [481, 482] used the SB system to introduce CD19-specific CARs to T cells and then applied the ZFN system to permanently delete TCR or HLA-A. Consequently, they demonstrated that these anti-CD19 CAR-T cells not only maintained anti-tumor activity but also ameliorated GvHD or evaded host T cell recognition, providing a basis for the preparation of universal CAR-T cells. Recently, Brown et al. successfully used the ZFN system to generate off-the-shelf, steroid-resistant CAR-T cells [317, 478] by electrotransferring anti-IL13Rα2 CAR into allogeneic T cells and using an Ad5/F35 vector-delivered ZFN to knock out the glucocorticoid receptor. After dexamethasone selection, these cells displayed dexamethasone-resistant effector activity without evidence for in vitro alloreactivity. Moreover, these cells were well tolerated and produced transient tumor reduction and/or tumor necrosis at the infusion site in four of the six treated research participants, indicating the safety and feasibility of such ZFN-modified cells for use as off-the-shelf allogeneic CAR-T cell products.

However, although TALEN and ZFN have the ability to mediate gene insertion, this feature is currently not applied in ACT due to certain shortcomings that need to be addressed, the most prominent of which is relative inefficiency. Additionally, compared to CRISPR/Cas9, TALENs and ZFNs are more expensive, difficult to handle, and time-consuming, restricting their broad applications, particularly for immune cell therapy, which requires the large-scale preparation of engineered immune cells. Therefore, in existing clinical studies of cell therapy, TALEN and ZFN are mainly used as auxiliary technologies for gene knockout.

Gene transduction is the basis and prerequisite of engineered cell therapy because it has a substantial impact on the quantity and quality of engineered cells. A good gene transduction method should be highly effective, safe, simple to use, and affordable, but no currently available technology possesses all of these characteristics. With the development of immune cell therapy, more immune cell types will be engineered. Consequently, there is a growing need for reliable transduction technology. Optimizing current techniques and investigating new ones that are highly effective, safe, simple to use, inexpensive, and capable of broad cell tropism are therefore urgently necessary. Multiple immune cell therapies will advance rapidly after the development of a sufficient number of transduction techniques suitable for different cell types and cargo features.

## Conclusion and perspectives

Here, we reviewed the current status of ACT techniques and the associated gene transduction strategies, along with the advantages, challenges, and potential solutions of these therapies and the progress of related research. Although it is clear that adoptive immune cell therapies have a strong potential to combat most types of human cancers, there is still room for improvement in terms of efficacy and safety for the currently available immunotherapies, and the technologies necessary to optimize immune cell therapies, such as gene transduction and editing, are currently not sufficiently powerful. A deeper understanding of immune cell biology and innovative, interdisciplinary strategies is required to overcome the obstacles currently impeding the potential of many immunotherapies.

CAR-T cell therapy is the most rapidly developing ACT and is becoming more commonly used in tumor therapy. Despite the success of CAR-T cell therapy in treating hematologic malignancies, fighting solid tumors has always been challenging due to a number of obstacles, including the lack of tumor-specific antigens, the ineffectiveness of CAR-T cells in trafficking to and infiltrating tumor sites, and the immunosuppressive TME that negatively affects CAR-T cell activation and persistence in tumor sites. While optimization of CAR-T cell therapy is still being studied, researchers agree that the CAR technology should be expanded to other immune cells with anti-tumor potential. Immune cells other than T cells, such as macrophages and NK cells, have thus been the focus of considerable attention and engineering attempts. Despite some advancement, these innovative immune cell therapies, particularly the recently developed CAR-NK and CAR-M cell therapies, have yet to achieve their full potential. The majority of these technologies face not only TME challenges but also cell-type-specific issues. For instance, CAR-NK cell therapy is laborious to use due to the short lifespan of NK cells and difficulty of gene transduction; CAR-M preparation is difficult, and efficacy in vivo is uncertain due to the highly plastic and difficult-to-expand nature of macrophages.

Although these new technologies offer renewed hope for the treatment of solid tumors, their efficacy is difficult to predict when used alone because they cannot circumvent the potently immunosuppressive TME. This setback must therefore be overcome by developing a technique to directly create a favorable TME or to allow engineered immune cells to remodel the unfavorable TME. The combination of engineered immune cells, particularly CAR-T cells, with small compounds and monoclonal antibodies appears to be a promising solution [483]. A considerable amount of preclinical data, as well as some limited clinical data, demonstrate how these combination tactics can enhance the TME and boost CAR-T cell efficacy. However, before such combinatorial techniques are extensively used in clinical practice, overlapping toxicity must be addressed. Considering the complexity and heterogeneity of the TME and the potential for overlapping toxicities from the combined administration of drugs, engineering strategies that can give immune cells the ability to remodel the unfavorable TME or confer them with intrinsic resistance to immunosuppression may be more promising than targeting only one specific pathway with pharmaceuticals [484]. Such strategies mainly include: (1) CAR optimization (i.e., replacing a costimulating motif or adding signal modules that promote cell survival); (2) forced expression of pro-inflammatory cytokines (such as IL-15) or knockout of inhibitory genes (such as PD-1) to increase the activity and persistence of engineered immune cells; and (3) enabling immune cells to stimulate host immune responses, such as the secretion of BiTE. All of these approaches have demonstrated potential in preclinical or clinical studies, but large-scale clinical data are still needed to confirm their efficacy.

**Fig. 9:**
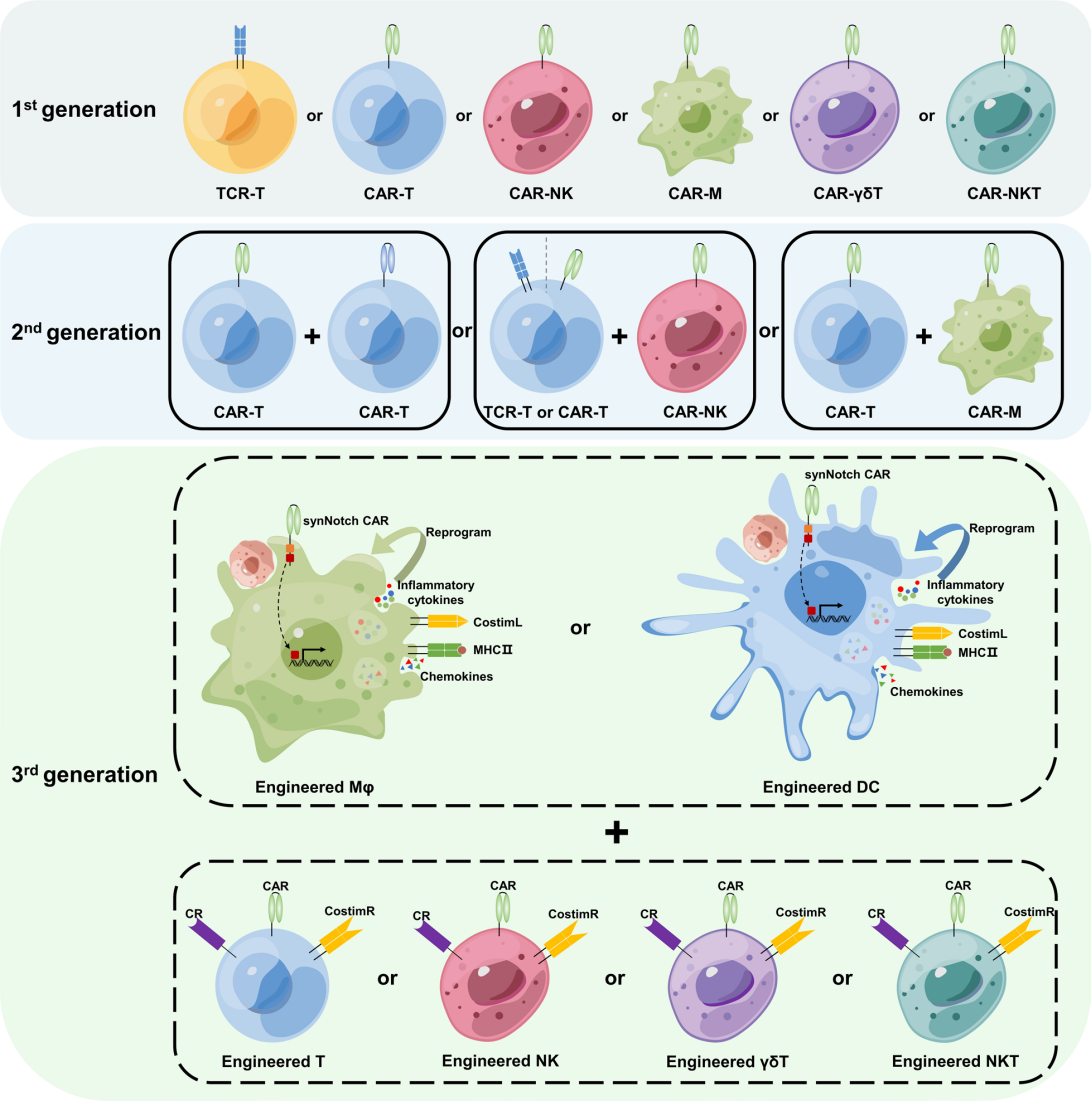
Current and future strategies of ACT in cancer treatment. Currently, adoptive cell therapy is still in its first generation and mainly relies on gene transduction technology to make certain immune cells express receptors that recognize tumor-associated antigens to kill tumor cells. Although many achievements have been made, single engineered immune cells face some challenges, such as poor persistence in vivo, antigen escape, and unpredictable side effects, indicating that adoptive cell therapy needs to be further developed. The second-generation adoptive cell therapy, which simply combines two types of CAR-engineered immune cells, has been preliminarily attempted and has demonstrated better anti-tumor effects. In the next step, we may fully exploit the unique properties of each type of manufactured immune cell, logically combine them to simulate a healthy immune coordination system, and artificially create a positive immune circuit. For example, macrophages and DCs serve as the commander of the immune system, with antigen presentation and a powerful ability to mobilize other immune cells. Combining them with engineered T cells, NK cells, γδT cells, or NKT cells to construct an artificial tumor-specific immune system may better overcome the limitations of current adoptive cell therapy techniques in solid tumors. This is a direction worth considering in the future. Mφ, macrophage; DC, dendritic cell; CostimL, costimulatory ligand; CostimR, costimulatory receptor; and CR, chemokine receptor

Another crucial factor to consider is the fact that most patients with advanced tumors have weakened immune systems as a result of repeated cycles of chemo-radiotherapy. A single type of engineered immune cell cannot significantly exert therapeutic efficacy by inducing endogenous protective immunity, even when equipped with immune excitation tools. In this instance, a positive feedback immune circuit created artificially by combining various types of engineered immune cells to simulate the healthy immune coordination mechanisms is a potential solution that may be considered in the future (Fig. 9). We believe that the strategies for achieving these immune coordinating circuits can be classified into two categories, which we have designated as generation 2 (G2) and generation 3 (G3) ACTs to differentiate them from the first generation of single-type immune cell therapies. The G2 ACTs can achieve complementary recognition or functional complementarity by combining the appropriate immune cell types based on their unique features. For instance, a combination of TCR-T and CAR-NK cells may not only destroy tumor cells with MHC-I, but can also enhance T cell-mediated anti-tumor immunity via NK-mediated recruitment of DCs [485]. When CAR-T cells were combined with CAR-M or CAR-NK cells, the interplay between naturally occurring costimulatory receptors/ligands and cytokines/receptors might create a positive synergistic network and hence increase the potency and durability of the anti-tumor immune response. In G3 ACTs, costimulatory receptor/ligand, cytokine, or chemokine interaction networks are artificially implanted in immune cells of interest to create simple but robust and stable innate-adaptive immune coordinating circuits that may improve the infiltration, killing capacity, and persistence of engineered immune cells. The costimulatory receptors/ligands employed can either be chimeric receptors and ligands that have been artificially modified for purposes such as enhancing persistence or naturally occurring molecules between APC and T cells, such as CD58/CD2, B7/CD28. There has been some advancement in this field thus far [486–488]. For instance, proof-of-concept research by Li et al. with anti-CD19 CAR-NK and anti-CD19 CAR-T cells showed that combining the two can increase anti-tumor cytotoxicity and persistence, enhance the general safety profile, and prevent tumor recurrence in the mouse xenografts model [487]. Similarly, we recently showed that the combination of CAR-T and CAR-M cells can significantly increase therapeutic efficacy against tumors [488]. It will likely be some time before such multi-cell combination ACTs are widely used in clinical settings, unless there is a breakthrough in the development of off-the-shelf universal cell products, as immediately obtaining multiple types of personalized engineered immune cells is difficult, expensive, and time-consuming. Considering this, future research should focus on developing universal cell products that not only reduce costs and preparation times but also realize the concept of selecting the best single type or combination of immune cells for precision treatment based on patient conditions. This immune cell circuit therapy is expected to become a reality and help in the treatment of solid tumors as immunotherapy theories and technologies advance.

### Supplementary Information

**Additional file 1.** Clinical trials of ACTs for cancer

## Data Availability

Not applicable.

## Abbreviations

ACT: Adoptive cell therapy
TCR: T cell receptor
CAR: Chimeric antigen receptor
TIL: Tumor-infiltrating lymphocyte
NK: Natural killer cell
M: Macrophage
NKT: Natural killer T
TME: Tumor microenvironment
CRS: Cytokine release syndrome
ORR: Objective response rate
CR: Complete response
FDA: US Food and Drug Administration
CRISPR/Cas9: Clustered regularly interspaced short palindromic repeats-associated protein 9
PDCD1: Programmed cell death 1
TALEN: Transcription activator-like effector nucleases
gp100: Glycoprotein 100
MHC: Major histocompatibility complex
TAA: Tumor-associated antigen
MAGE: Melanoma-associated antigen family
Tn: Naïve T cell
Tscm: Stem cell-like memory T cell
Tcm: Central memory T cell
siRNA: Small interfering RNA
scFv: Single-chain variable fragment
NT: Neurotoxicity
SUPRA: Split, universal, and programmable
PSMA: Prostate-specific membrane antigen
PSCA: Prostate stem cell antigen
synNotch: Synthetic Notch receptor
SWIFF: Small molecule protease-based regulation strategy
BiTE: Bispecific T cell engager
TRUCK: T cells redirected for universal cytokine-mediated killing
HER2: Epidermal growth factor receptor 2
LAT: Linker for activation of T cells
LINK: Logic-gated intracellular network
FITC: Fluorescein isothiocyanate
FcγR: Fragment crystallizable gamma receptor
ADCC: Antibody-dependent cellular cytotoxicity
rtTA: Reverse tetracycline transcriptional activator
HCC: Hepatocellular carcinoma
ECM: Extracellular matrix
GD2: Disialoganglioside
BTLA: B and T lymphocyte attenuator
HVEM: Herpes virus entry mediator
MDSC: Myeloid-derived suppressor cell
DC: Dendritic cell
HIF-1α: Hypoxia-inducible factor 1α
ODD: Oxygen-dependent degradation domain
HRE: Hypoxia response element
APC: Antigen-presenting cell
LOV2: Light-oxygen-voltage domain 2
GM-CSF: Granulocyte-macrophage colony-stimulating factor
UCNP: Upconversion nanoplates
CEA: Carcinoembryonic antigen
CAIX: Carbonic anhydrase IX
OV: Oncolytic virus
GvHD: Graft versus host disease
HvGR: Host versus graft rejection
BCMA: B cell maturation antigen
HDAC: Histone deacetylase
DNMT: DNA methyltransferase
AP1: Activator protein 1
FAO: Fatty acid oxidation
OxPhos: Oxidative phosphorylation
ROS: Reactive oxygen species
PB: Peripheral blood
UCB: Umbilical cord blood
hESC: Human embryonic stem cell
iPSC: Induced pluripotent stem cell
BCL: B cell lymphoma
AML: Acute myeloid leukemia
PBMC: Peripheral blood mononuclear cell
AAV: Adeno-associated viral
TAM: Tumor-associated macrophage
MMP: Matrix metalloproteinases
MerTK: Mer receptor tyrosine kinase
HSC: Hematopoietic stem cell
HIV-1: Human immunodeficiency virus-1
cPPT: Central polypurine tract
SB: Sleeping beauty transposon system
PB: PiggyBac transposon system
LNP: Lipid nanoparticles
gRNA: Guide RNA
DSB: Double-strand break
NHEJ: Non-homologous end joining
HDR: Homology-directed repair
LAG-3: Lymphocyte activation gene-3
